# Synthesis, activity evaluation, and pro-apoptotic properties of novel 1,2,4-triazol-3-amine derivatives as potent anti-lung cancer agents

**DOI:** 10.1080/14756366.2019.1636044

**Published:** 2019-07-09

**Authors:** Xian-yu Sun, Chun-yan Zhong, Qing-qing Qiu, Zhen-wang Li, Mei-yu Liu, Xin Wang, Cheng-hao Jin

**Affiliations:** a Department of Pharmacy, College of Animal Science and Technique, Heilongjiang Bayi Agricultural University, Daqing, China;; b Department of Biochemistry and Molecular Biology, College of Life Science and Technology, Heilongjiang Bayi Agricultural University, Daqing, China

**Keywords:** Synthesis, anti-cancer, lung cancer, apoptosis, BCL-2

## Abstract

In this study, a series of 4,5-bis(substituted phenyl)-4*H*-1,2,4-triazol-3-amine compounds was designed, synthesised, and evaluated to determine their potential as anti-lung cancer agents. According to the results of screening of lung cancer cell lines A549, NCI-H460, and NCI-H23 *in vitro*, most of the synthesised compounds have potent cytotoxic activities with IC_50_ values ranging from 1.02 to 48.01 µM. Particularly, compound 4,5-bis(4-chlorophenyl)-4*H*-1,2,4-triazol-3-amine (**BCTA**) was the most potent anti-cancer agent, with IC_50_ values of 1.09, 2.01, and 3.28 µM against A549, NCI-H460, and NCI-H23 cells, respectively, meaning many-fold stronger anti-lung cancer activity than that of the chemotherapeutic agent 5-fluorouracil. We also explored the effects of **BCTA** on apoptosis in lung cancer cells by flow cytometry and western blotting. Our results indicated that **BCTA** induced apoptosis by upregulating proteins BAX, caspase 3, and PARP. Thus, the potential application of compound **BCTA** as a drug should be further examined.

## Graphical Abstract

**Figure:**
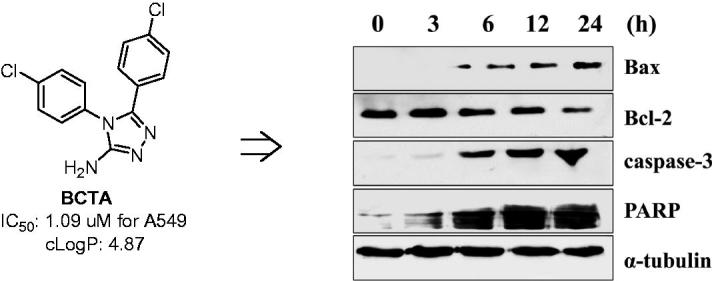

An outstanding compound **BCTA** (4,5-bis(4-chlorphenyl)-4H-1,2,4-triazol-3-amine) was found to possess more potent anticancer activity against several lung cancer cells than the reference drugs 5-FU. The effects of **BCTA** on apoptosis and necrosis in A549 cells were analysed by Hoechst/propidium iodide (PI) double staining and early and late apoptosis were analysed by Annexin V/PI flow cytometry. Further Western bloting results revealed that **BCTA** could induce cell apoptosis by down-regulting Bcl-2 protein.

## 1. Introduction

According to one study, lung cancer ranks first in cancer mortality worldwide, and the number of cases is currently increasing. In 2018, nearly 1,972,000 new lung cancer cases and 1,748,000 lung cancer deaths occurred globally^1^. Therefore, the discovery and/or synthesis of novel anti-cancer agents is necessary for eliminating this urgent global health problem. The discovery of novel anti-cancer agents with highly effective modes of action against tumours is an important and challenging task.

Triazole and its derivatives have a broad range of biological and pharmacological properties, such as anti-bacterial, anti-human immunodeficiency virus^2^, anti-microbial^3^, anti-fungal^4^, anti-viral^5^, and anti-cancer effects^6–14^. For example, Abdou *et al*. designed and synthesised the triazole structure of amino-thiadiazole/thiadiazide-phosphate esters and studied the anti-cancer characteristics on 10 cancer cell lines^7^. Although triazole derivatives can inhibit the proliferation of several cell types, their anti-cancer mechanism of action remains unclear.

**Figure 1.**
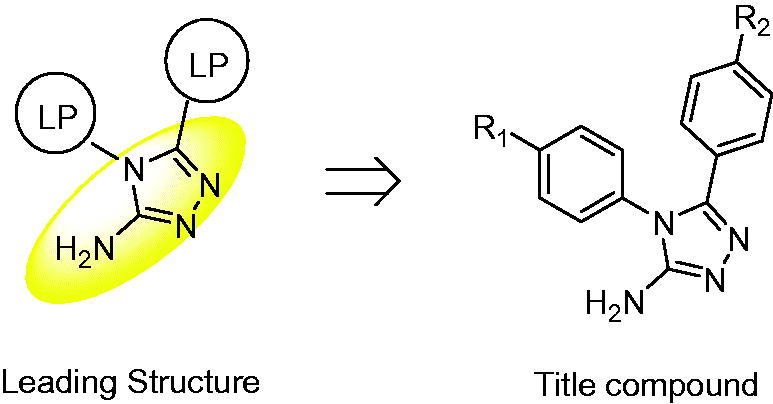
Design of the title compound. LP: Lipophilic group; the core is highlighted in yellow.

In our continuing efforts to develop novel anti-lung cancer candidate compounds, we became interested in 3-aminotriazole. The common use of the 3-aminotriazole skeleton in many synthetic drugs indicates its potential as a new pharmacophore. In this study, we examined the anti-cancer properties of amino triazole derivatives to achieve better activity by appropriate structure modification (Figure 1). Amino triazole was present in the core of the designed compounds, with substituted phenyl groups introduced at positions 4 and 5. We hypothesised that a reasonable lipid–water partition coefficient could be obtained via introduction of aromatic groups into the pharmacophore, thus enabling the target compound to easily penetrate the plasma membrane and enter the cell. Meanwhile, introduction of heterocyclic groups into amino triazole has been reported to increase its *in vitro* activity^15^. Thus, 4,5–(4-substituted)diphenyl-4*H*-1,2,4-triazol-3-amine derivatives were designed and prepared as potential anti-lung cancer agents. The incidence of lung cancer is related to various factors, and the mechanism of its induction is complex and diverse, whereas inhibition of lung cancer formation and metastasis is mainly regulated by induction of apoptosis. Expression of some apoptotic proteins is altered during apoptosis^16^^,^^17^. In this study, the anti-cancer mechanisms of action of different compounds were evaluated from the perspective of apoptosis. The anti-cancer activities of these compounds were evaluated *in vitro*, and their structure–activity relations and anti-cancer mechanisms of action were investigated too.

## 2. Material and methods

### 2.1. Chemistry

Chromatography was conducted on silica gel 60, 200 mesh (Qingdao Haiyang, China), under high pressure supplied by an aquarium air pump (SB-2800). Analytical thin-layer chromatography (TLC) was performed on precoated silica gel 60 F254 plates (Qingdao Haiyang, China). The plates were visualised using UV light and/or iodine. Melting points (m.p.) were determined in open capillary tubes and were uncorrected. Chemical shifts are reported in ppm relative to tetramethyl silane. The structures of all the synthesised compounds were confirmed by ^1^H, ^13 ^C nuclear magnetic resonance (NMR) spectroscopy and mass spectra. Mass spectra were recorded on an HP1100 LC/MSD electrospray ionisation mass spectrometer (Agilent Technologies, Santa Clara, CA, USA) and NMR spectra on an AV-300 FT-NMR spectrometer (Bruker BioSpin AG, Billerica, MA, USA). All the chemicals were of analytical grade. The detailed preparation process of compounds **4a–4j** is described below.

### 2.2. 4-Substituted-N-(4-chlorophenyl)benzamide (1a–1e) and 4-Substituted-N-phenylbenzamide (1f–1j)

A mixture of aromatic acid (10 mmol), aromatic amine (10 mmol), DMAP (10 mmol), and EDCI (10 mmol) in *N*,*N*-dimethyl formamide (DMF; 40 ml) was heated to 60 °C while stirring for 1 h. TLC revealed the completion of the reaction. After cooled, the mixture was filtered and washed with water, producing a light-white solid in 76.0***–***89.9% yield.

### 2.3. 4-Substituted-N-(4-chlorophenyl)benzothioamide (2a–2e) and 4-Substituted-N-phenylbenzothioamide (2f–2j)

A mixture of **1** (5 mmol), and Lawson reagent (5 mmol) in toluene (20 ml) was heated to reflux. After 24 h, the end of the reaction was confirmed by TLC. After cooling, the reaction mixture was filtered and evaporated to give a light-yellow solid as a crude product. The compounds obtained were pure enough for the following step.

### 2.4. N-Substituted phenyl-N'-amino substituted benzamidine (3a–3j)

A mixture of **2** (5 mmol) and hydrazine hydrate (5 mmol) in ethanol (30 ml) was heated to 80 °C. After 1.5 h, the end of the reaction was confirmed by TLC. The reaction mixture was poured into cooled water (30 ml), and the precipitates was filtered and evaporated to give a light-white solid in 58.2***–***77.6% yield.

### 2.5. General synthetic procedure for target compounds (4a–4j)

In the presence of NaHCO_3_, a mixture of compound **3** (1.0 mmol) and BrCN (1.1 mmol) in water (30 ml) was stirred at –5 °C 12–16 h. TLC showed that most of the starting material was reacted. The reaction mixture was extracted with dichloromethane (2 × 50 ml). The combined organic layers were dried over anhydrous MgSO_4_, filtered, and evaporated; the residue was purified by silica gel column chromatography with ethyl acetate and methanol (8:1). The yields, m.p., and spectral data for each compound are shown below.

#### 2.5.1. 4-(4-Chlorophenyl)-5-phenyl-4H-1,2,4-triazol-3-amine (4a)

Yield 80%, m.p. 168–170 °C; ^1^H-NMR (300 MHz, CDCl_3_), *δ*: 2.05 (s, 2H, –NH_2_), 7.22–7.52 (m, 9H, Ar-H), ^13 ^C-NMR (300 MHz, CDCl_3_), *δ*: 126.43, 127.83, 128.60, 128.63, 129.65, 130.83, 131.93, 136.22. ESI-MS m/z 271 (M + 1), 273 (M + 3), 274 (M + 4), calculated for [C_14_H_11_ClN_4_] 270.

#### 2.5.2. 4-(4-Chlorophenyl)-5-(4-bromophenyl)-4H-1,2,4-triazol-3-amine (4b)

Yield 79%, m.p. 248–250 °C; ^1^H-NMR (300 MHz, CDCl_3_), *δ*: 4.59 (s, 2H, –NH_2_), 7.21–7.53 (m, 8H, Ar-H), ^13 ^C-NMR (300 MHz, CDCl3), *δ*: 128.57, 129.09, 130.91, 131.85, 136.34. ESI-MS m/z 350 (M + 1), 351 (M + 2), calculated for [C_14_H_10_BrClN_4_] 349.

#### 2.5.3. 4,5-Bis(4-chlorophenyl)-4H-1,2,4-triazol-3-amine (4c)

Yield 86%, m.p. 247–248 °C; ^1^H-NMR (300 MHz, CDCl_3_), *δ*: 4.76 (s, 2H, –NH_2_), 7.21–7.53 (m, 8H, Ar-H), ^13 ^C-NMR (300 MHz, CDCl_3_), *δ*: 125.38, 128.58, 128.85, 128.88, 130.86, 132.14, 135.53, 136.20, 148.95, 154.80. ESI-MS m/z 307 (M + 2), 308 (M + 3), calculated for [C_14_H_10_Cl_2_N_4_] 305.

#### 2.5.4. 4-(4-Chlorophenyl)-5-(4-fluorophenyl)-4H-1,2,4-triazol-3-amine (4d)

Yield 88%, m.p. 208–212 °C; ^1^H-NMR (300 MHz, CDCl_3_), *δ*: 4.31 (s, 2H, –NH_2_), 6.95–7.52 (m, 8H, Ar-H), ^13 ^C-NMR (300 MHz, CDCl_3_), *δ*: 115.66, 115.96, 122.81, 128.67, 129.74, 129.85, 130.83, 131.89, 136.26, 161.67, 164.99. ESI-MS m/z 289 (M + 1), 291 (M + 3), 292 (M + 4), calculated for [C_14_H_10_FClN_4_] 288.

#### 2.5.5. 4-(4-Chlorophenyl)-5-(4-(methylsulfonyl)phenyl)-4H-1,2,4-triazol-3-amine (4e)

Yield 83%, m.p. >280 °C; ^1^H-NMR (300 MHz, CDCl_3_), *δ*: 3.05 (s, 3H, –CH_3_), 4.63 (s, 2H, –NH_2_), 7.24–7.88 (m, 8H, Ar-H), ^13 ^C-NMR (300 MHz, CDCl_3_), *δ:* 43.65, 127.63, 127.96, 130.31, 130.69, 132.66, 132.98, 134.64, 140.90, 147.54, 156.82. ESI-MS m/z 349 (M + 1), 351 (M + 3), calculated for [C_15_H_13_O_2_N_4_] 348.

#### 2.5.6. 4-Phenyl-5-(4-bromophenyl)-4H-1,2,4-triazol-3-amine (4f)

Yield 86%, m.p. 268–271 °C; ^1^H-NMR (300 MHz, CDCl_3_), *δ*: 4.58 (s, 2H, –NH_2_), 7.22–7.56 (m, 8H, Ar-H), ^13 ^C-NMR (300 MHz, CDCl_3_), *δ*: 123.64, 126.04, 127.25, 129.02, 130.63, 131.69, 133.63. ESI-MS m/z 317 (M + 1), 318 (M + 2), calculated for [C_14_H_11_BrN_4_] 316.

#### 2.5.7. 4-Phenyl-5-(4-chlorophenyl)-4H-1,2,4-triazol-3-amine (4g)

Yield 83%, m.p. 243–246 °C; ^1^H-NMR (300 MHz, CDCl_3_), *δ*: 4.65 (s, 2H, –NH_2_), 7.20–7.55 (m, 9H, Ar-H), ^13 ^C-NMR (300 MHz, CDCl_3_), *δ*: 125.41, 127.28, 128.77, 128.86, 130.18, 130.66, 133.44, 135.46. ESI-MS m/z 271 (M + 1), 273 (M + 3), calculated for [C_14_H_11_ClN_4_] 270.

#### 2.5.8. 4-Phenyl-5-(4-fluorophenyl) -4H-1,2,4-triazol-3-amine (4h)

Yield 81%, m.p. 207–211 °C; ^1^H-NMR (300 MHz, CDCl_3_), *δ*: 4.64 (s, 2H, –NH_2_), 6.91–7.55 (m, 9H, Ar-H), ^13 ^C-NMR (300 MHz, CDCl_3_), *δ*: 115.42, 115.71, 123.46, 127.30, 128.81, 129.57, 129.68, 129.95, 130.52, 130.85, 133.78, 149.15, 154.77, 161.48, 164.79. ESI-MS m/z 255 (M + 1), calculated for [C_14_H_11_FN_4_] 254.

#### 2.5.9. 4-Phenyl-5-(4-methyphenyl)-4H-1,2,4-triazol-3-amine (4i)

Yield 76%, m.p. 245–250 °C; ^1^H-NMR (300 MHz, CDCl_3_), *δ*: 1.23–1.28 (t, *J* = 6 Hz, 9 Hz, 3H, –CH_3_), 4.54 (s, 2H, −NH_2_), 7.04–7.53 (m, 8H, Ar-H), ^13 ^C-NMR (300 MHz, CDCl_3_), *δ*: 21.24, 124.31, 127.35, 127.59, 129.08, 129.74, 130.38, 134.04, 139.25. ESI-MS m/z 251 (M + 1), calculated for [C_15_H_14_N_4_] 250.

#### 2.5.10. 4-Phenyl-5–(4-(trifluoromethyl)phenyl)-4H-1,2,4-triazol-3-amine (4j)

Yield 86%, m.p. 222–225 °C; ^1^H-NMR (300 MHz, CDCl_3_), *δ*: 4.90 (s, 2H, –NH_2_), 7.27–7.58 (m, 9H, Ar-H), ^13 ^C-NMR (300 MHz, CDCl_3_), *δ*: 125.40, 125.45, 127.24, 127.61, 130.30, 130.50, 130.77, 133.51, 148.64, 155.71. ESI-MS m/z 305 (M + 1), 306 (M + 2), calculated for [C_14_H_11_CF_3_N_4_] 304.

### 2.6. The cell viability assay

The three lung cancer cell lines, A549, NCI-H460, and NCI-H23, and three normal cell lines, normal lung IMR-90 cells, normal liver L-02 cells, and normal gastric GES-1 cells, were acquired from the American Type Culture Collection (Manassas, VA, USA). The cells were cultured in RPMI-1640 supplemented with 10% of foetal bovine serum, 100 U/mL penicillin, and 100 µg/mL streptomycin (Gibco, Grand Island, NY, USA), at 5% CO_2_ and 37 °C. The cells were monitored daily and maintained at 80% cell density^18^.

Cell viability was determined by the MTT assay. Lung cancer cells (A549, NCI-H460, and NCI-H23) and normal human lung IMR-90 cells, liver L-02, and gastric GES-1 cells were harvested during the logarithmic phase of growth. All the cells were seeded in 96-well plates at 10^4^ cells/well and then treated with various concentrations (0.1, 0.3, 1.0, 3.0, or 30 µmol/mL) of 5-FU or one of amino triazole derivatives for 24 h. The MTT solution (15 µL; 5 mg/mL) was added into each well, and the cells were cultured for 4 h at 37 °C. The supernatants were removed, and resolved with 100 µL of DMSO, and the cells were shocked for 10 min. The optical density of the samples was measured at 490 nm on a microplate luminometer. Cell viability was expressed as the percentage change in absorbance compared to the control values.

### 2.7. Cell apoptosis analysis

Apoptosis and necrosis were analysed by Hoechst/ propidium iodide (PI) double staining and early and late apoptosis were analysed by Annexin V/PI flow cytometry. A549 cells were seeded in 6-well plates (10^5^ cells/well) and treated with 2 µM 5-FU or one of amino triazole derivatives for 0, 3, 6, 12, and 24 h. After that, the cells were collected and washed once in phosphate-buffered saline (PBS). The cells were resuspended in 195 µL of binding buffer, and dual staining was performed with 3 µL of Hoechst or Annexin V and 2 µL of PI, and the cells were incubated for 20 min at room temperature in the dark. Apoptotic cells were analysed using the EVOS FL Auto Cell Imaging System (Thermo Fisher Scientific, Inc., Waltham, MA, USA) and a flow cytometer (Beckman Coulter, Inc., Brea, CA).

### 2.8. Western blotting analysis

A549 cells were washed with PBS and lysed in cell extraction buffer (1 M 4-[2-hydroxyethyl]-1-piperazineethanesulfonic acid [HEPES] pH 7.0, 5 M NaCl, 0.5% Triton X-100, 10% of glycerol, 20 mM β-mercaptothion, 20 mg/mL 4–(2-aminoethyl)benzenesulfonyl fluoride hydrochloride [AEBSF], 0.5 mg/mL pepstatin, 0.5 mg/mL leupeptin, and 2 mg/mL aprotinin). Cell lysates were centrifuged for 30 min at 12,000 × *g* and 4 °C. Aliquots of the lysates were separated by sodium dodecyl sulphate polyacrylamide gel electrophoresis in a 10% gel and transferred onto nitrocellulose membranes. The membranes were blocked for 1 h with 5% skim milk at room temperature, incubated for 12 h with primary antibodies: mouse monoclonal antibodies against α-tubulin, BAX, BCL-2, cleaved poly[ADP-ribose] polymerase (cle-PARP), and then cleaved caspase 3 at 4 °C (all from Santa Cruz Biotechnology, Inc., Dallas, TX). The membranes were washed with Tris-buffered saline supplemented with 0.1% Tween 20 (TBST) and incubated with secondary antibodies: a goat anti-rabbit IgG antibody or goat anti-mouse IgG antibody at room temperature for 2 h. The Pierce ECL reagent (Rockford, IL, USA) was added, and the bands were detected on an Amersham Imager 600 (GE, Little Chalfont, UK) and quantified in the ImageJ software (NIH, Bethesda, MD, USA). Relative protein expression in the treated cells was compared to that in the control group. The endogenous control was α-tubulin.

### 2.9. Statistical analysis

This procedure was performed by one-way analysis of variance in SPSS 19.0 software (SPSS, Inc., Chicago, IL, USA), and the results are presented as the means ± SD. A value of *p* < .05 was assumed to indicate statistical significance.

## 3. Results and discussion

### 3.1. Chemistry

**Scheme 1.**
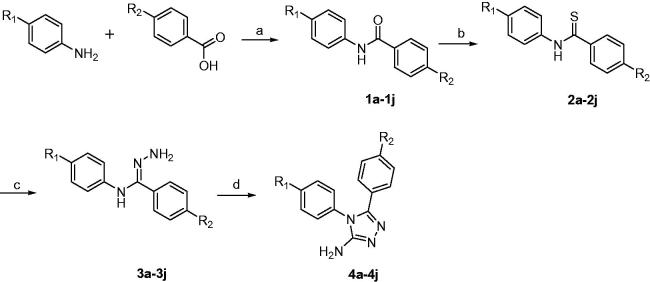
Synthesis of target compounds **4a–4j**. (a) EDCI, DMAP, DMF, 60 °C; (b) Lawesson’s reagent, methylbenzene, 100 °C; (c) N_2_H_4_ H_2_O, EtOH, 80 °C; (d): NaHCO_3_, BrCN, –5 °C.

**Scheme 2.**
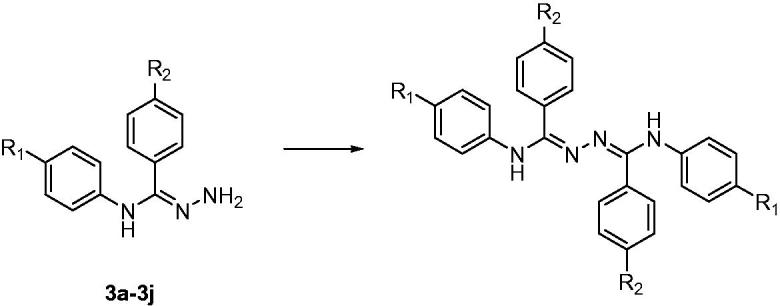
Structural change in compounds **3a–3j**.

A series of 4,5-(substituted phenyl)-4*H*-1,2,4-triazol-3-amine derivatives **(4a–4j)** was designed and synthesised according to Scheme 1. Compounds **1a–1j** were prepared by reacting *p*-substituted aniline and *p*-substituted benzoic acid with EDCI and DMAP in dimethylformamide (DMF)^19^. The oxygen atoms of compounds **1a–1j** were converted to sulphur atoms by Lawesson’s reagent in refluxing toluene to produce compounds **2a–2j**^20^. Compounds **3a–3j** were obtained in a 70–80% yield by means of hydrazine hydrate at 80 °C. Compounds **4a–4j** were prepared by reacting compounds **3a–3j** with cyanogen bromide (BrCN) and sodium bicarbonate in water at –5 °C. If the reaction temperature was >0 °C, a structural change was detected, as shown in Scheme 2, and the molecular weight indicated that di-substitution had occurred. Nonetheless, this change was avoided by maintaining the reaction mixture at –5 °C.

### 3.2. The viability of lung cancer cells treated with 4a–4j

The synthesised compounds were subjected to the cytotoxicity assay by the 3–(4,5-dimethylthiazol-2-yl)-2,5-diphenyltetrazolium bromide (MTT) method as described previously^21^. IC_50_ is defined as the drug concentration causing a 50% reduction of cell number compared with that of untreated control. In the first screening, we evaluated the effects of the compounds on the growth of three lung cancer cell lines (A549, NCI-H460, and NCI-H23). Their anti-cancer activities were measured and are presented in Table 1. The results were averages of three separate measurements. 5-Fluorouracil (5-FU) served as a positive control.

**Table 1.** IC_50_ (µM) values of 5-FU and of amino triazole derivatives towards lung cancer cells.
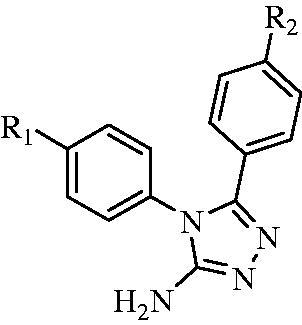

Comp.	*R*_1_	*R*_2_	Lung cancer cells			cLogP
			A549	NCI-H460	NCI-H23	
**5-FU**	–	–	28.24 ± 1.53	61.56 ± 3.62	33.68 ± 1.34	–
**4a**	Cl	H	1.23 ± 0.08	8.52 ± 0.41	6.94 ± 0.36	4.10
**4b**	Cl	Br	1.02 ± 0.05	7.27 ± 0.42	6.06 ± 0.44	5.14
**4c (BCTA)**	Cl	Cl	1.09 ± 0.06	2.01 ± 0.11	3.28 ± 0.15	4.87
**4d**	Cl	F	2.32 ± 0.18	7.59 ± 0.43	7.91 ± 0.48	4.32
**4e**	Cl	SO_2_CH_3_	45.02 ± 2.83	>100	45.43 ± 2.79	2.84
**4f**	H	Br	48.01 ± 3.86	32.04 ± 1.85	42.66 ± 3.78	4.45
**4g**	H	Cl	3.16 ± 0.45	8.77 ± 0.55	9.04 ± 0.74	4.27
**4h**	H	F	3.64 ± 0.32	10.98 ± 1.02	9.61 ± 0.81	3.74
**4i**	H	CH_3_	11.32 ± 1.37	15.69 ± 1.56	18.04 ± 1.64	3.97
**4j**	H	CF_3_	31.22 ± 2.54	70.36 ± 4.69	37.81 ± 2.63	4.48

IC_50_: Each value was averaged by three parallel groups of eight repeats and calculated using a SigmaPlot software. cLogP: It was obtained by analysing the chemical properties of structures in Chemdraw software.

As presented in Table 1, most compounds significantly inhibited the growth of three lung cancer cell lines in a dose-dependent manner. Seven compounds were found to be more potent than 5-FU. The most potent compound **4c** (4,5-bis(4-chlorophenyl)-4*H*-1,2,4-triazol-3-amine, **BCTA**) exerted cytotoxicity 10- to 20-fold more potent than that of 5-FU with IC_50_ of 1.09 µM towards A549 cells, 2.01 µM towards NCI-H460 cells, and 3.28 µM towards NCI-H23 cells, respectively, at 24 h.

**Figure 2.**
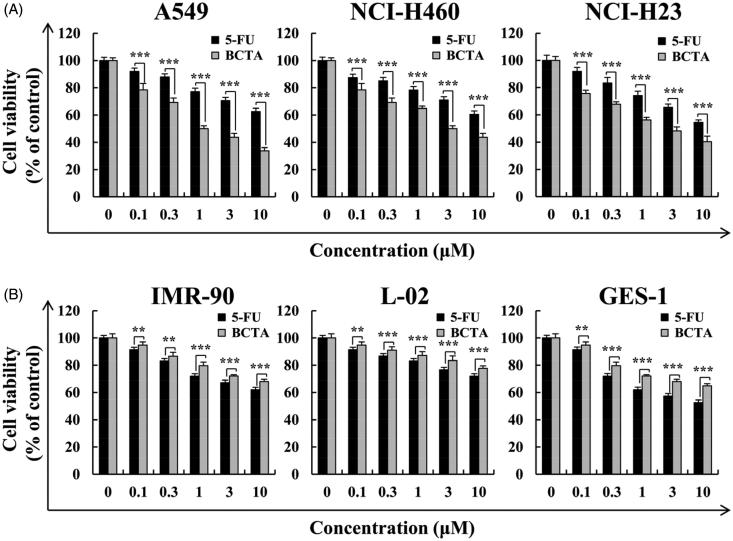
Effects of **BCTA** on cell viability in human lung cancer cells. (A) Three human lung cancer cell lines (A549, NCI-H460, and NCI-H23) and (B) three normal cell lines (IMR-90, L-02, and GES-1) were treated with different concentrations (0.1, 0.3, 1.0, 3.0, or 30 μM) of 5-FU or BCTA for 24 h. Error bars indicate the means ± SD of three independent experiments (*p < .05, **p < .01, ***p < .001 indicate significant differences).

Furthermore, the cytotoxic effects of **BCTA** were significantly weaker (as compared to treatment with 5-FU) towards normal lung IMR-90, liver L-02, and gastric GES-1 cells (Figure 2). A549 cells showed the highest sensitivity to these compounds, and thus we choose these cells for further analysis.

### 3.3. BCTA-induced apoptosis in A549 cells

**Figure 3.**
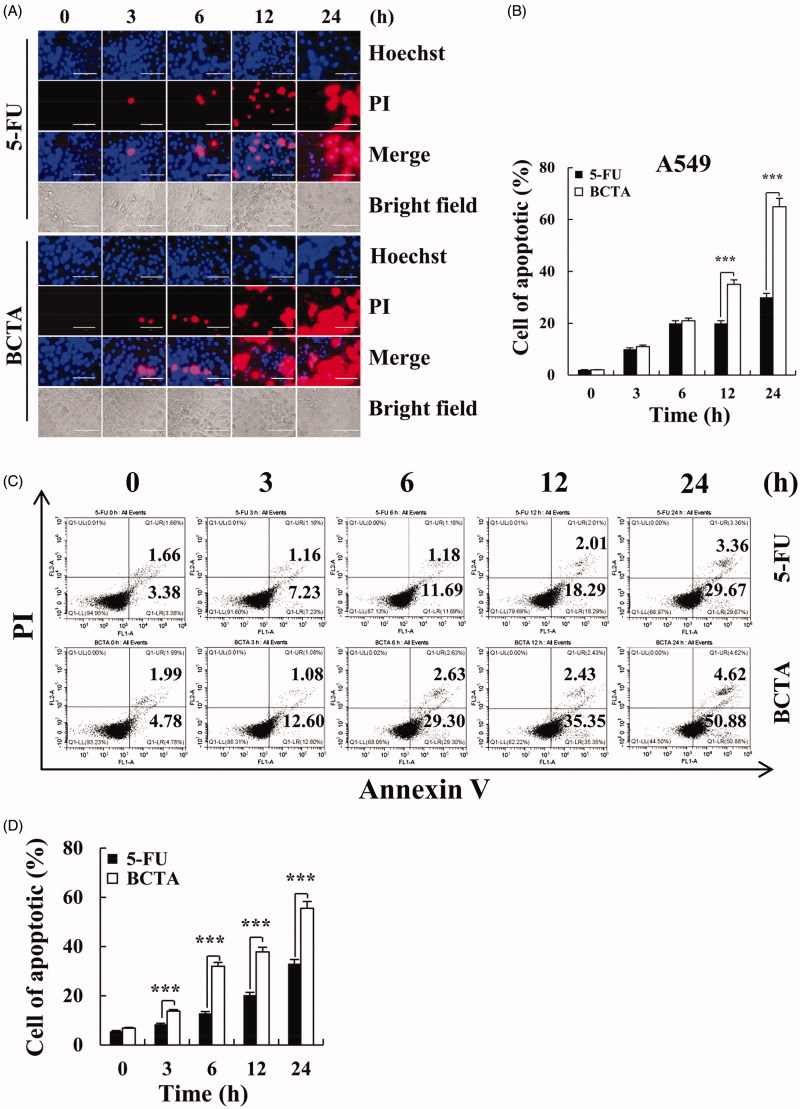
Induction of apoptosis and necrosis by **BCTA** in A549 cells. (A) A549 cells were treated with 2 μmol/mL of **BCTA** or 5-FU for different periods (0, 3, 6, 12, or 24 h) and stained with the Hoechst/PI solution. The morphology and fluorescence intensity were examined under a fluorescence microscope (magnification: ×200). (B) Quantification of fluorescence intensity. (C) A549 cells were treated with 2 μmol/mL **BCTA** or 5-FU for 0, 3, 6, 12, or 24 h, stained with the annexin V/PI solution, and analysed by flow cytometry. (D) Quantification of the percentages of apoptotic cells. Error bars indicate the means ± SD of three independent experiments (*p < .05, **p < .01, ***p < .001 indicate significant differences).

To determine whether amino triazole derivatives induce A549 cell apoptosis, we conducted Hoechst/PI double staining and an annexin V/PI flow-cytometric assay to elucidate the mechanism. As depicted in Figure 3(A,B), the experimental group was significantly better than the control treatment 5-FU. Particularly, at 12 and 24 h, **BCTA** treatment resulted in a 64% apoptosis rate, which was higher than the rate caused by 5-FU: 35%. According to the results of flow cytometry, the apoptosis rate afforded by **BCTA** was 55.5% (Figure 3(C,D)), and the results were nearly consistent with those of Hoechst/PI staining.

### 3.4. Western blotting result of BCTA in A549 cells

**Figure 4.**
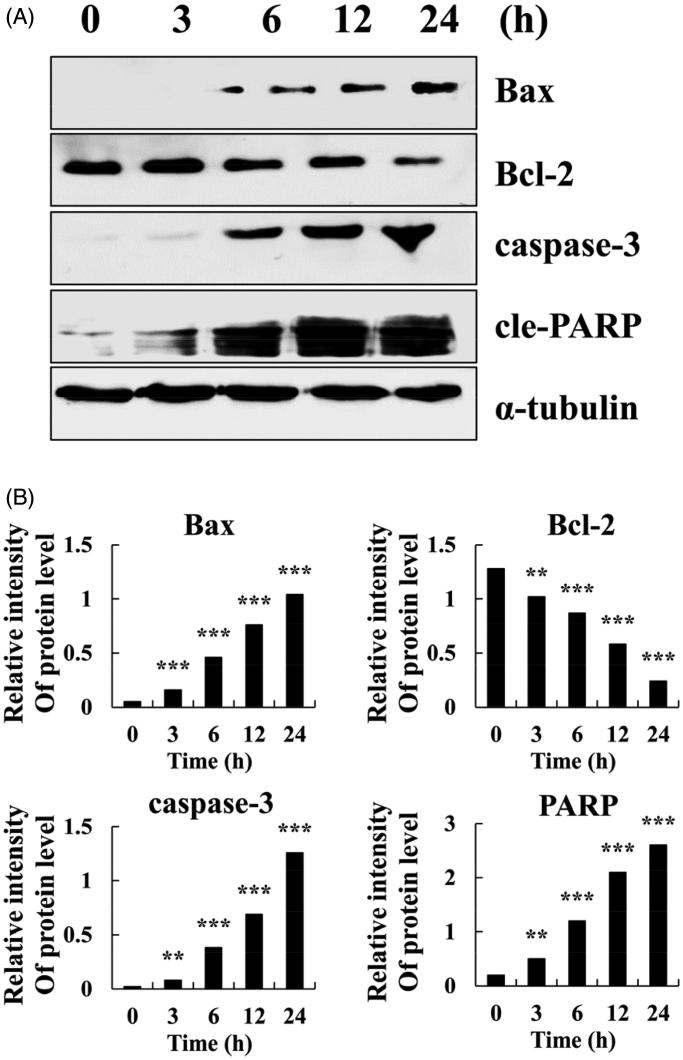
Apoptotic effects of **BCTA** on A549 cells. (A) Effects of **BCTA** on the expression levels of BAX, BCL-2, caspase 3, and cleaved PARP in A549 lung cancer cells were analysed by western blotting and (B) densitometric quantification. *p < .05, **p < .01, ***p < .001 relative to the control group (0 h). BCL-2, B-cell lymphoma 2; BAX, BCL-2-associated agonist of cell death; cle-PARP, cleaved poly(adenosine diphosphate ribose) polymerase.

For further evaluation of intrinsic apoptosis mechanism, **BCTA** was tested for its effect on the expression of four critical peptides. Results of the Westen blotting analysis were expressed as the relative tation of the specific band compared with the internal reference. **BCTA** clearly increased the protein expression levels of BAX, caspase 3, and PARP and decreased the protein expression levels of BCL-2 (Figure 4(A,B)) in a time dependence manner (3–24 h). The results suggested that **BCTA** induced apoptosis in A549 cells by up-regulating the mitochondrial apoptotic pathways.

### 3.4. Discussion

Amino triazole derivatives have been reported to possess a broad range of biological and pharmacological properties, such as anti-bacterial, anti-human immunodeficiency virus, anti-microbial, anti-fungal, anti-viral, and anti-cancer effects. Nevertheless, their anti-cancer mechanism of action remains unclear. Because this is our first study on the compounds carrying the 3-aminotriazole moiety as potential anti-cancer agents, we initially designed 10 compounds to roughly identify general structural features required for anti-cancer cytotoxicity. This study uncovered a part of the mechanism of action, and the relation between its structure and anti-cancer activity through pharmacological analysis. The results showed that the compounds induced apoptosis in tumour cells and inhibited cell proliferation by changing the expression of relevant proteins to achieve anti-cancer activity. The strength of this anti-cancer activity was found to be related to various substituents. According to Table 1, the anti-neoplastic activities of four chlorobenzene-substituted compounds (**4a**–**4d**) were significantly better than those of the 5-phenyl-4*H*-1,2,4-triazol-3-amine structures (**4f**–**4j**). Our results showed that their toxicity (particularly that of **BCTA**) towards normal cells was less than that of 5-FU in the control group.

It is well known that the activity of drugs is mainly determined by their structure. One of the main factors is the lipid–water distribution coefficient (LogP value). LogP is an interpretation of the lipid phase and water phase equilibrium ratio of compounds. It can also be employed to determine pharmacological parameters. An increase in the lipid–water distribution coefficient indicates increased solubility of the compound in fat; in contrast, a decrease in this coefficient means increased solubility in water. The solubility of a compound in fatty substances present in the body is known as the hydrophobicity of that compound, whereas the solubility of a compound in water is known as hydrophilicity of that compound. The human body absorbs hydrophilic compounds more easily because of its high water content^22^. Nonetheless, the cell membrane has a phospholipid bilayer structure that is lipophilic in nature. Thus, if a hydrophilic component of a compound is too large, it cannot enter the cell. Therefore, anti-cancer compounds should be lipophilic to some extent. The extent of drug absorption may also be affected by the magnitude of the LogP value. Hence, an optimal LogP value is necessary to allow a material to easily enter the cell membrane and participate in biological functions. The optimal value for cLogP is approximately 5^23^. Due to the complexity of factors affecting drug activity, a drug needs a suitable cLogP value, but an appropriate cLogP value does not guarantee a good activity. As shown in Table 1, compound **4b** and **BCTA** seem to be two promising structures.

In-depth analysis of the structure–activity relationship of these structures revealed that most of the tested compounds exerted an cytotoxic activity against lung cancer cells (IC_50_ 1.02–48.01 µM). The compounds containing a halogen atom on the phenyl ring showed better activity than did the compounds without a halogen atom. We also found that the Cl atom contributed more to the toxicity towards lung cancer cells than Br and F atoms did. As shown in Table 1, **BCTA** showed the strongest activity against lung cancer cell lines A549, NCI-H460, and NCI-H23 with IC_50_ values of 1.09–3.28 µM. Similarly, **4g** (4-phenyl, 5-(*p*-Cl)phenyl) exerted a stronger activity against lung cancer cells than did **4f** (4-phenyl, 5-(*p*-Br)phenyl) and **4h** (4-phenyl, 5-(*p*-F)phenyl). Meanwhile, compounds **4a** (5-phenyl), **4b** (5-(*p*-Br)phenyl), and **4d** (5-(*p*-F)phenyl) also posse good activity against three lung cancer cells. This level of growth inhibition was multiple stronger than that of 5-FU against lung cancer cells. IC_50_ values of derivatives **4e** (4–(4-chlorophenyl), 5-(*p*-SO_2_CH_3_)phenyl) and **4h** (4-phenyl, 5-(*p*-CH_3_)phenyl) against lung cancer cells were higher (worse) than IC_50_ of the control group (5-FU). Thus, no significant difference was observed in the activity between the compounds containing electron-donating groups and those containing electron-withdrawing groups.

As discussed above, most of the synthesised compounds with a halogen atom at the *para*-position of the 5-phenyl ring manifested a strong toxic activity against lung cancer cells. Finally, compound **BCTA** was the most potent cytotoxic agent, with IC_50_ values of 1.09, 2.01, and 3.28 µM against A549, NCI-H460, and NCI-H23 cells, respectively. These IC_50_ values were many times stronger than those of the reference agent 5-FU. So, compound **BCTA** was further screened its apoptosis-inducing capability and possible action mechanism.

Apoptosis is a conserved programmed cell death mechanism involved in the elimination of cancer cells^24^^,^^25^. Intrinsic apoptosis depends on factors released from the mitochondria and is also known as mitochondrial apoptosis^26^. The mitochondrial pathway is regulated by BCL-2 family members, which include pro-apoptotic protein BAX and anti-apoptotic protein BCL-2. Caspase 3 is a critical enzyme in apoptosis because it cleaves several essential cellular proteins such as PARP^27^. Many anti-cancer agents induce the intrinsic apoptotic pathway, which is characterised by activation of caspase 3 and cleavage of PARP^28^. The present study indicates that amino triazole derivatives significantly induced the apoptosis of A549 cells by up-regulating BAX, down-regulating BCL-2, and activating caspase 3 and PARP. These findings suggest that amino triazole derivatives regulate BCL-2 and BAX expression and activate the mitochondrial apoptotic cascade in lung cancer cells.

## 4. Conclusions

In summary, we synthesised novel amino triazole derivatives, particularly 4,5-bis (4-chlorophenyl)-4*H*-1,2,4-triazol-3-amine (**BCTA**), and investigated the anti-cancer molecular mechanisms of action of **BCTA**. **BCTA** induced apoptosis in human lung cancer cells by triggering the apoptosis pathways. On the basis of our observations, **BCTA** is a promising candidate for the development of anti-lung cancer drugs.

## Supplementary Material

Supplemental Material
