# Supplementary material for: Synthesis, activity evaluation, and pro-apoptotic properties of novel 1,2,4-triazol-3-amine derivatives as potent anti-lung cancer agents
Source: J Enzyme Inhib Med Chem. 2019 Jul 9;34(1):1210–7. doi: 10.1080/14756366.2019.1636044 (PMC6691921; doi:10.1080/14756366.2019.1636044)
Supplement: Supplemental Material [file IENZ_A_1636044_SM0386.pdf]

Synthesis, Activity Evaluation, and Apoptosis Induction of Novel 1,2,4-triazol-3-amine Derivatives as Potent Anti-lung Cancer Agents

Xian-yu Sun[1,*], Chun-yan Zhong[1], Qing-qing Qiu[1], Zhen-wang Li[1], Mei-yu Liu[1], Xin Wang[1], Cheng-hao Jin[2,*]

College of Animal Science and Technique, Heilongjiang Bayi Agriculture University, Daqing 163319, Heilongjiang, PR China

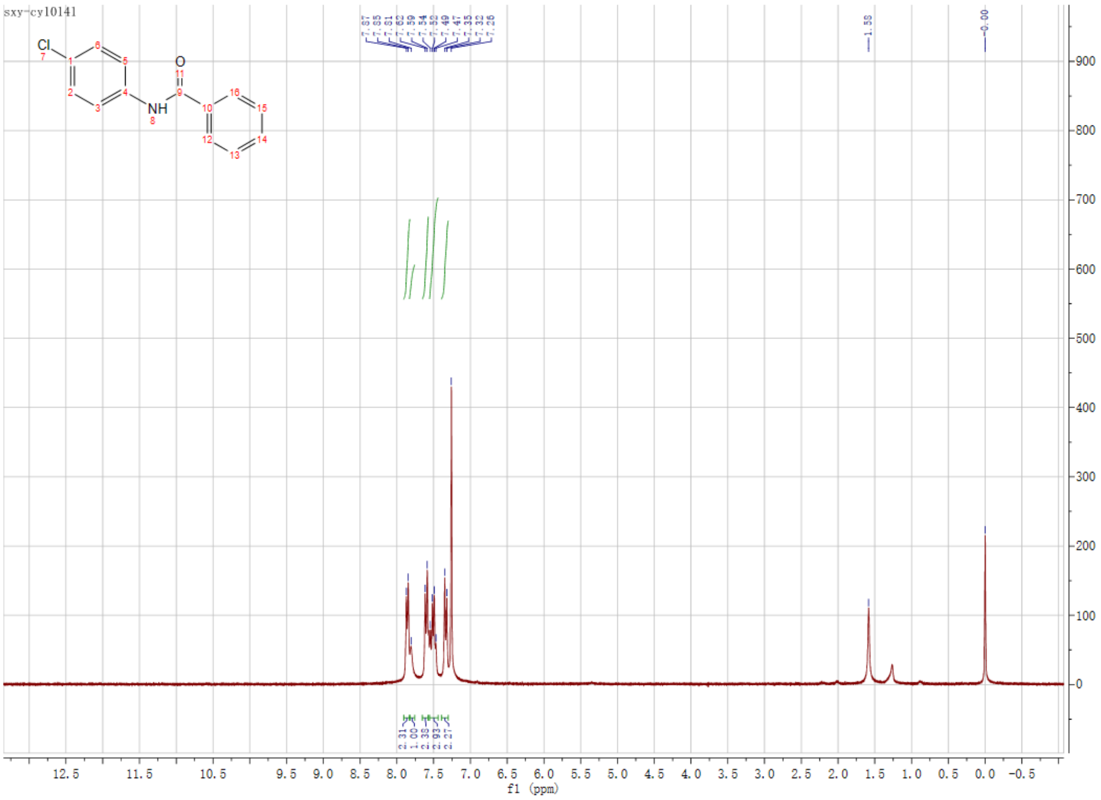

^{1}H-NMR of *N*-(4-chlorophenyl)benzamide (**1a)**

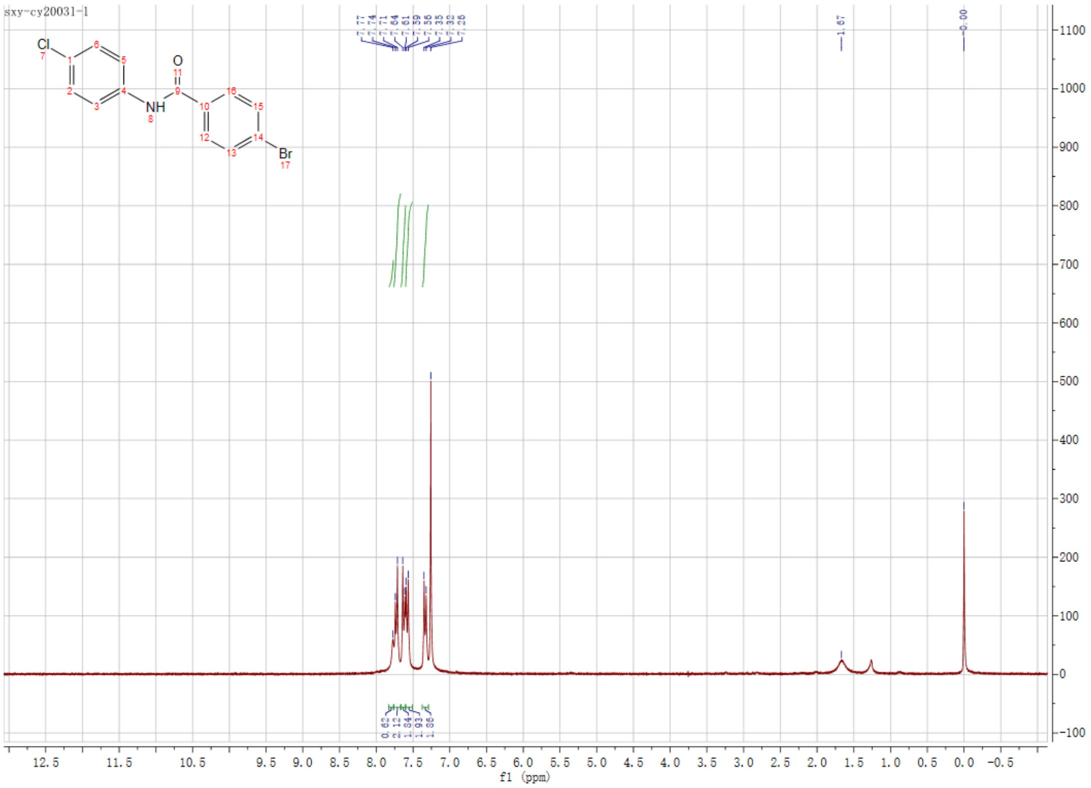

^{1}H-NMR of 4-bromo-*N*-(4-chlorophenyl)benzamide (**1b)**

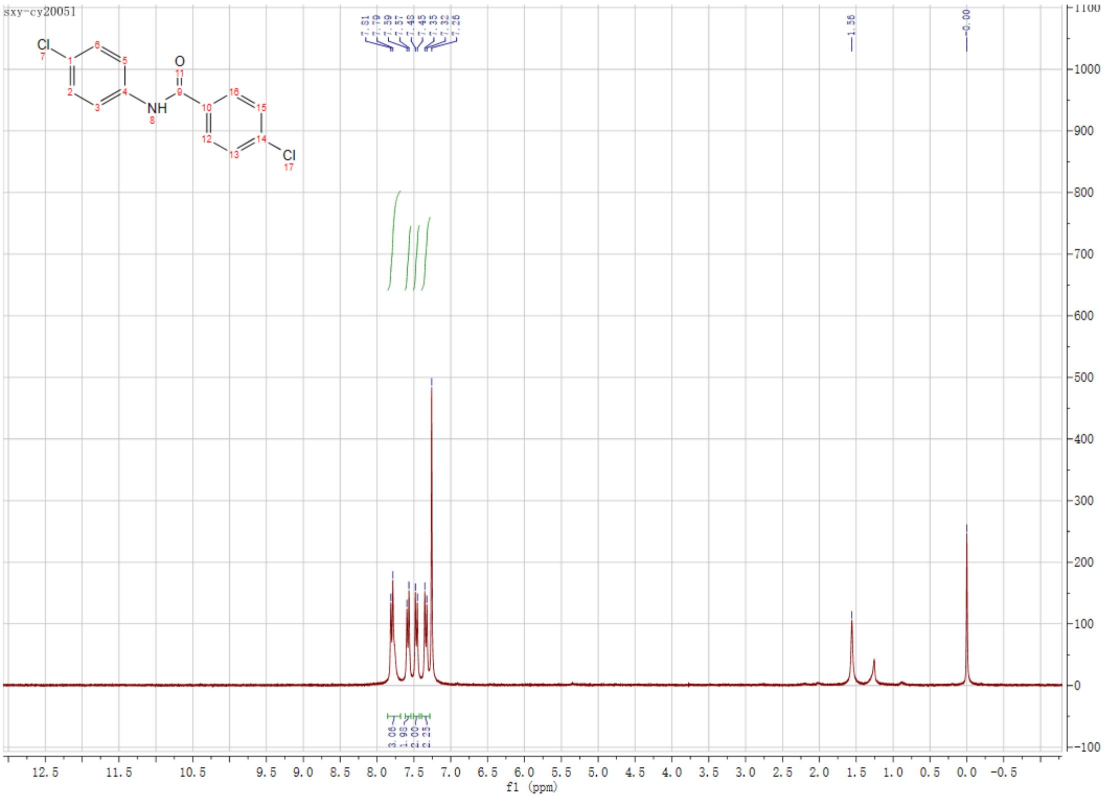

^{1}H-NMR of 4-chloro-*N*-(4-chlorophenyl)benzamide (**1c)**

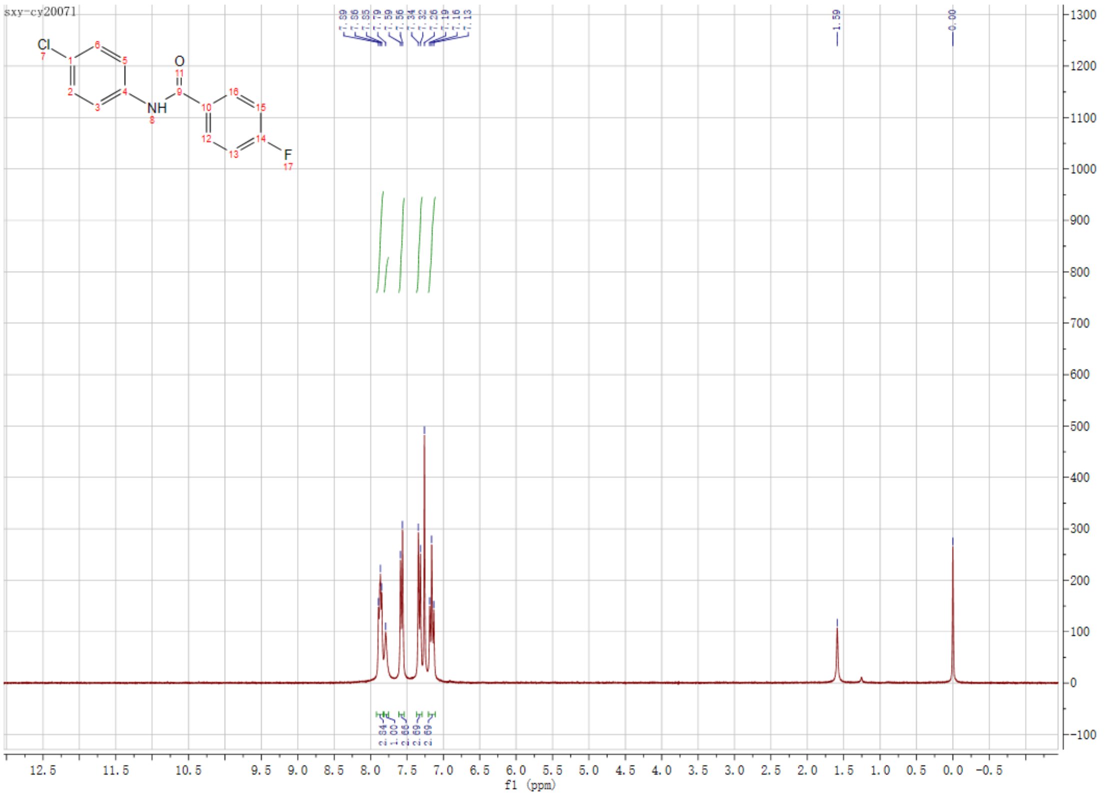

^{1}H-NMR of *N*-(4-chlorophenyl)-4-fluorobenzamide (**1d)**

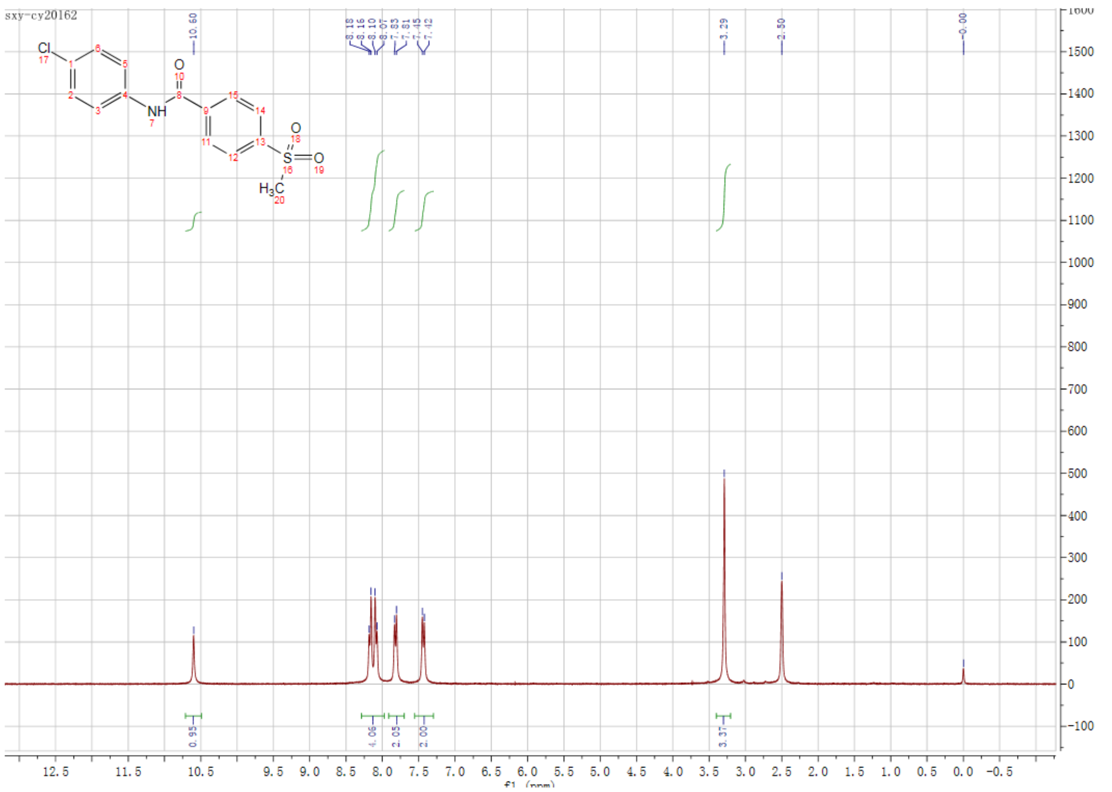

^{1}H-NMR of *N*-(4-chlorophenyl)-4-(methylsulfonyl)benzamide (**1e**)

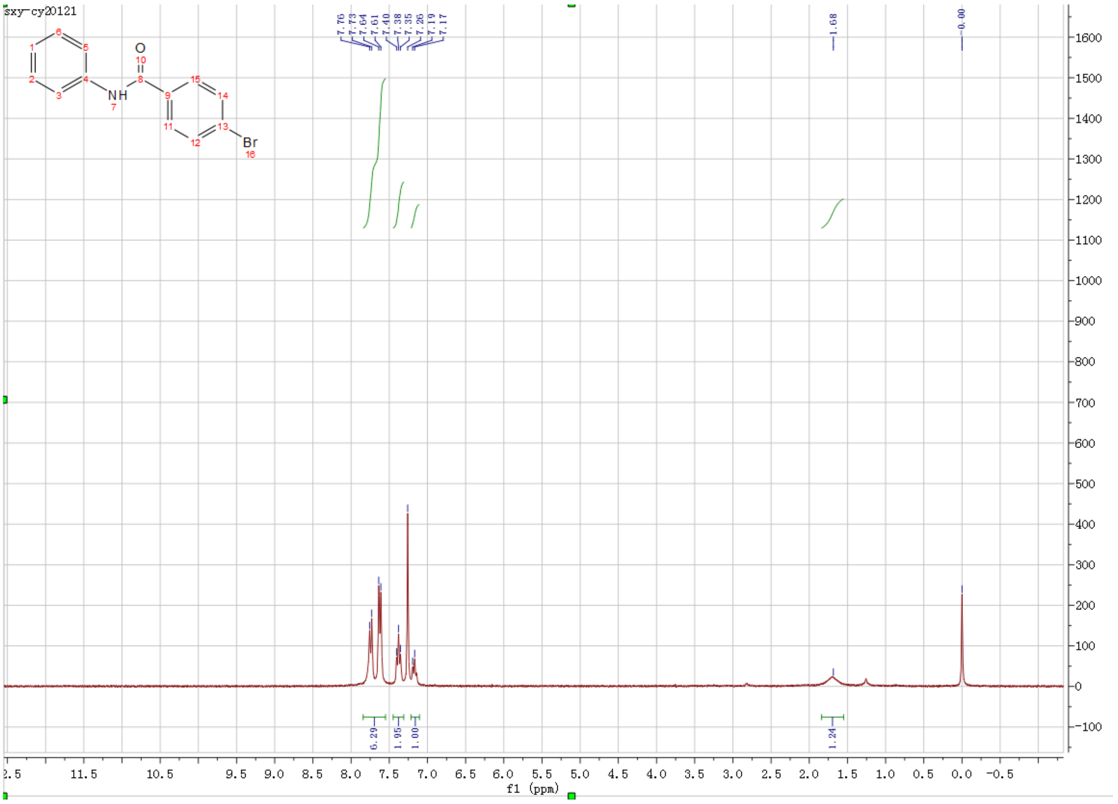

^{1}H-NMR of 4-bromo-*N*-phenylbenzamide (**1f**)

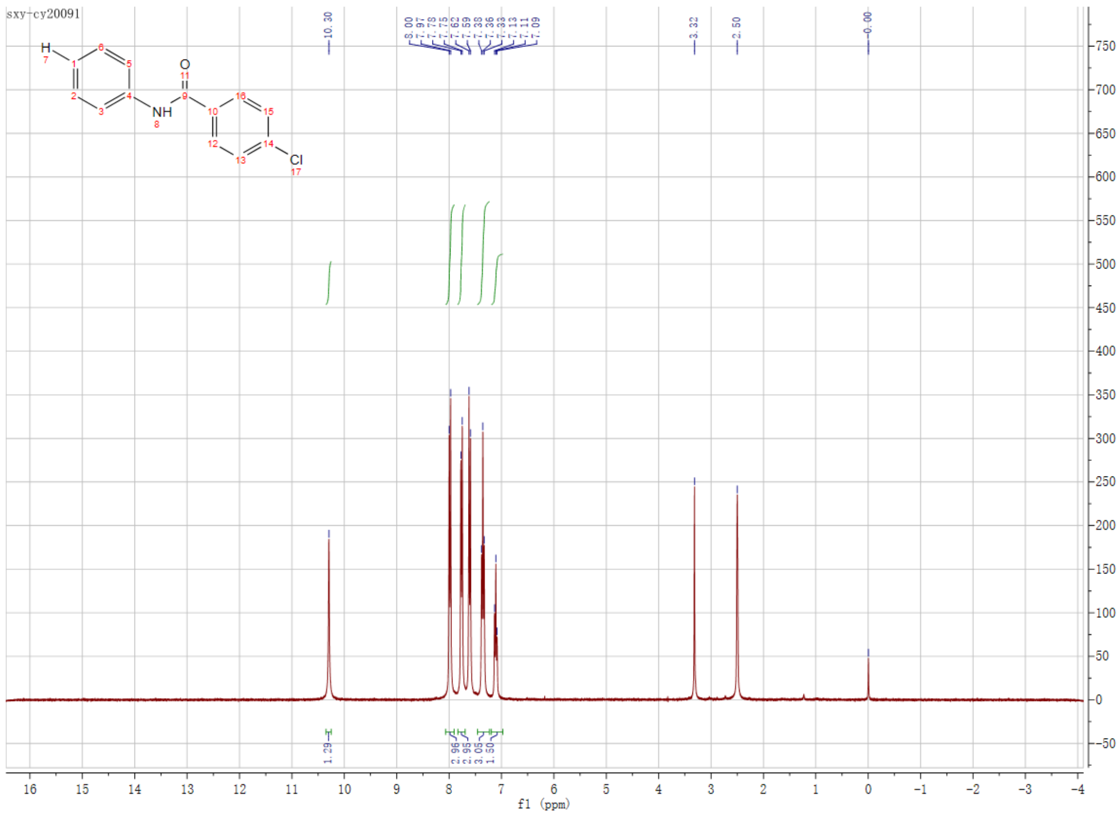

^{1}H-NMR of 4-chloro-*N*-phenylbenzamide (**1g**)

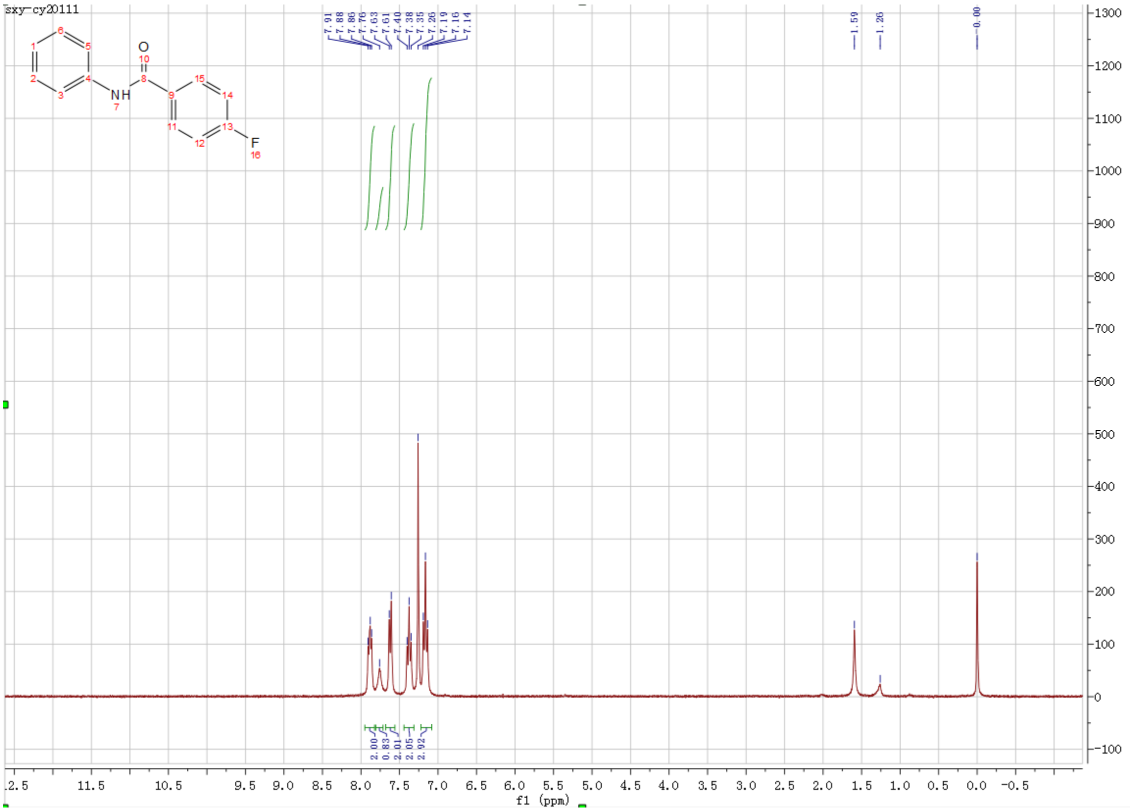

^{1}H-NMR of 4-fluoro-*N*-phenylbenzamide (**1h**)

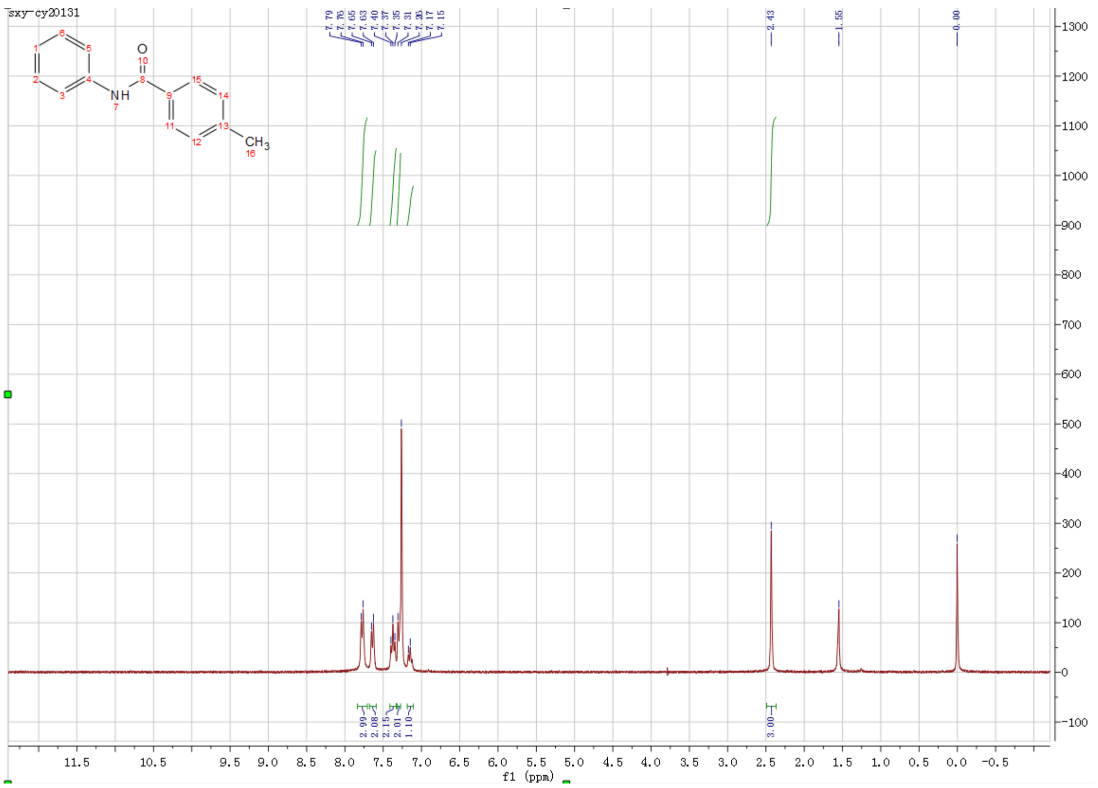

^{1}H-NMR of 4-methyl-*N*-phenylbenzamide (**1i)**

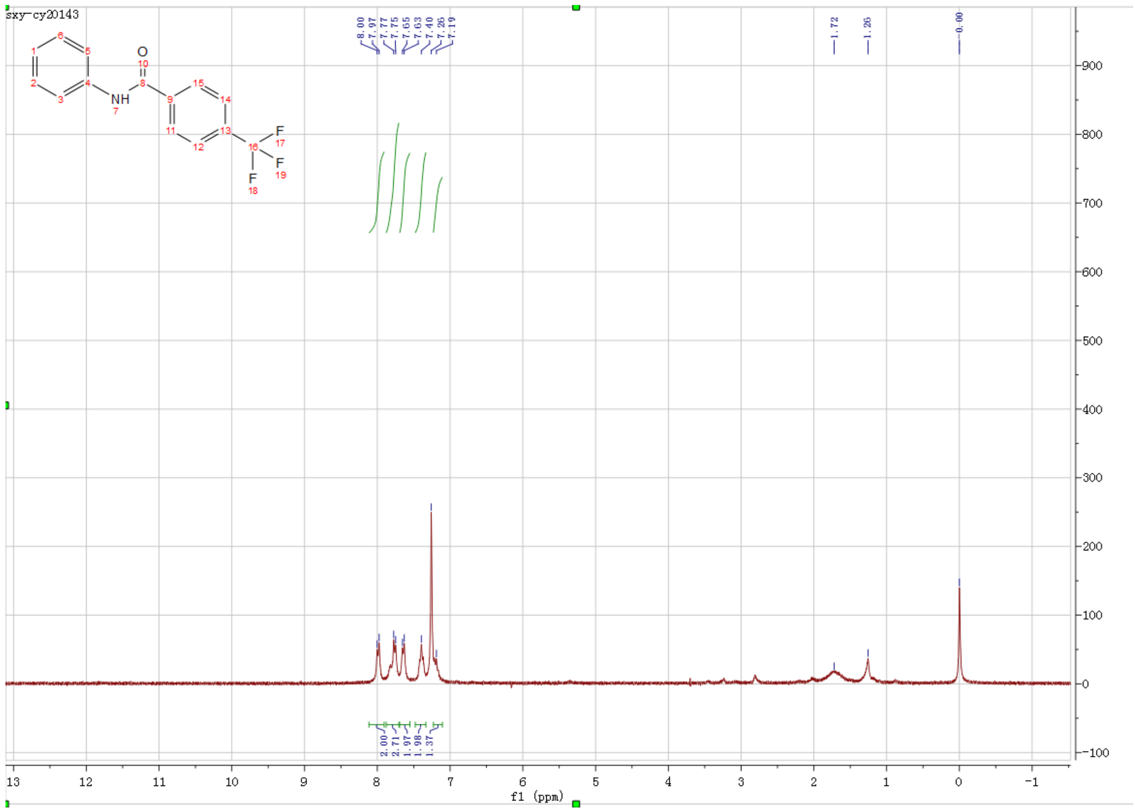

^{1}H-NMR of 4-(trifluoromethyl)-*N*-phenylbenzamide (**1j)**

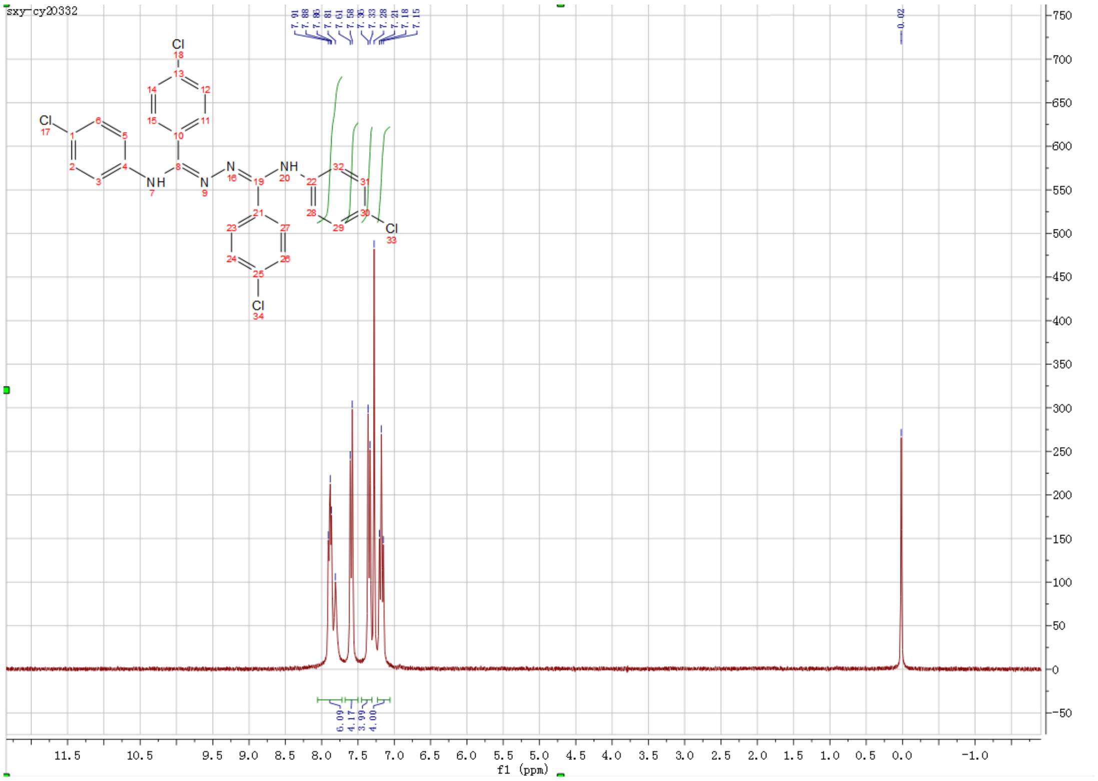

^{1}H-NMR of dimerization

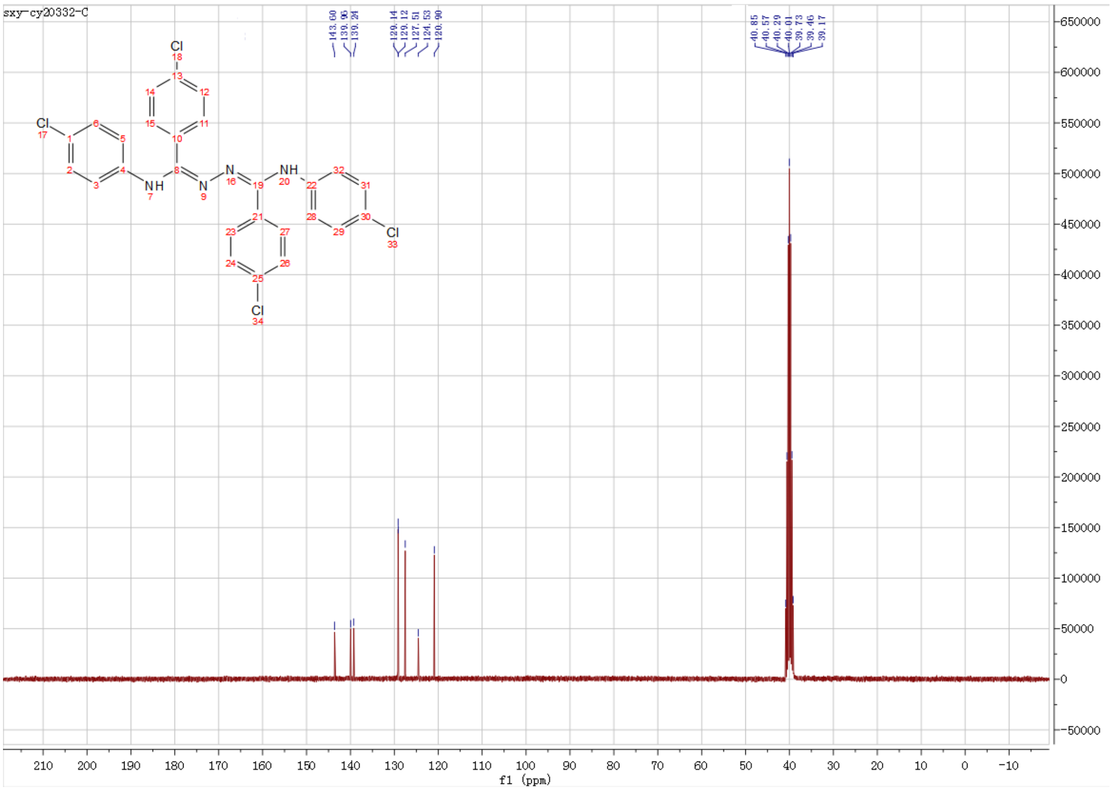

^{13}C-NMR of dimerization

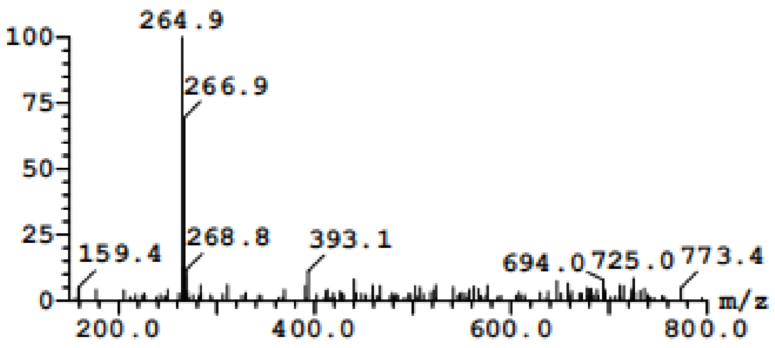

ESI-MS of dimerization

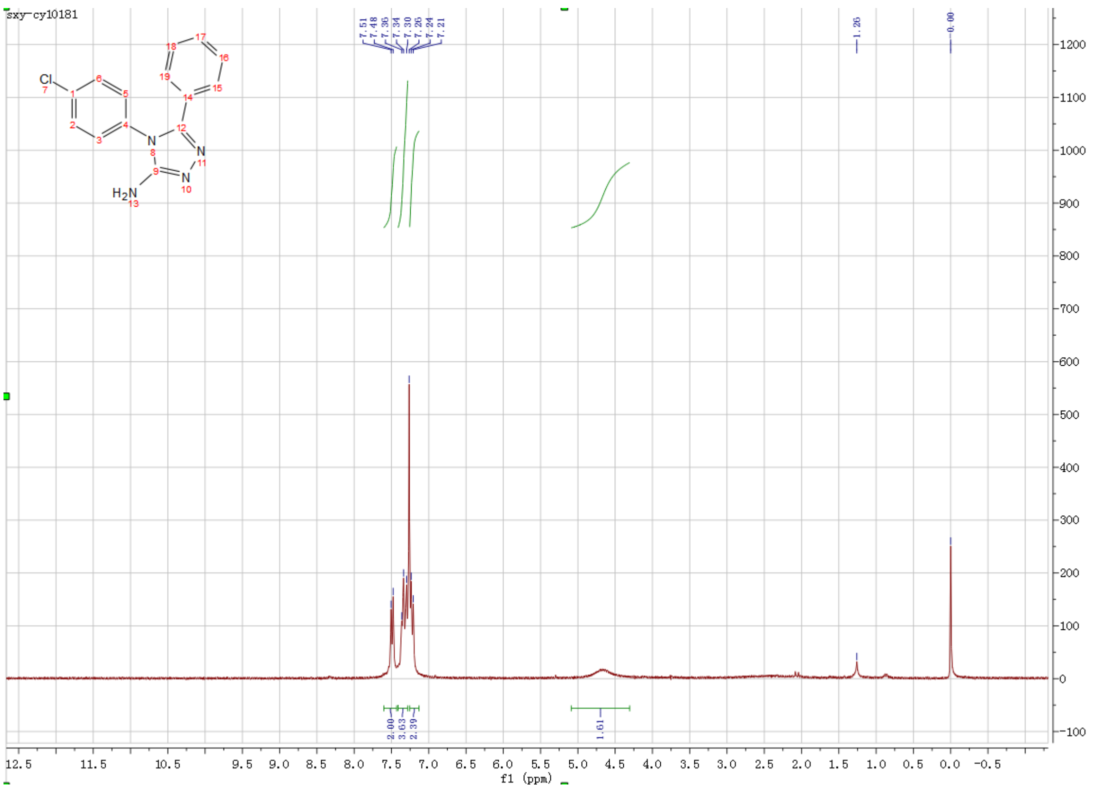

^{1}H-NMR of 4-(4-chlorophenyl)-5-phenyl-4*H*-1,2,4-triazol-3-amine **(4a)**

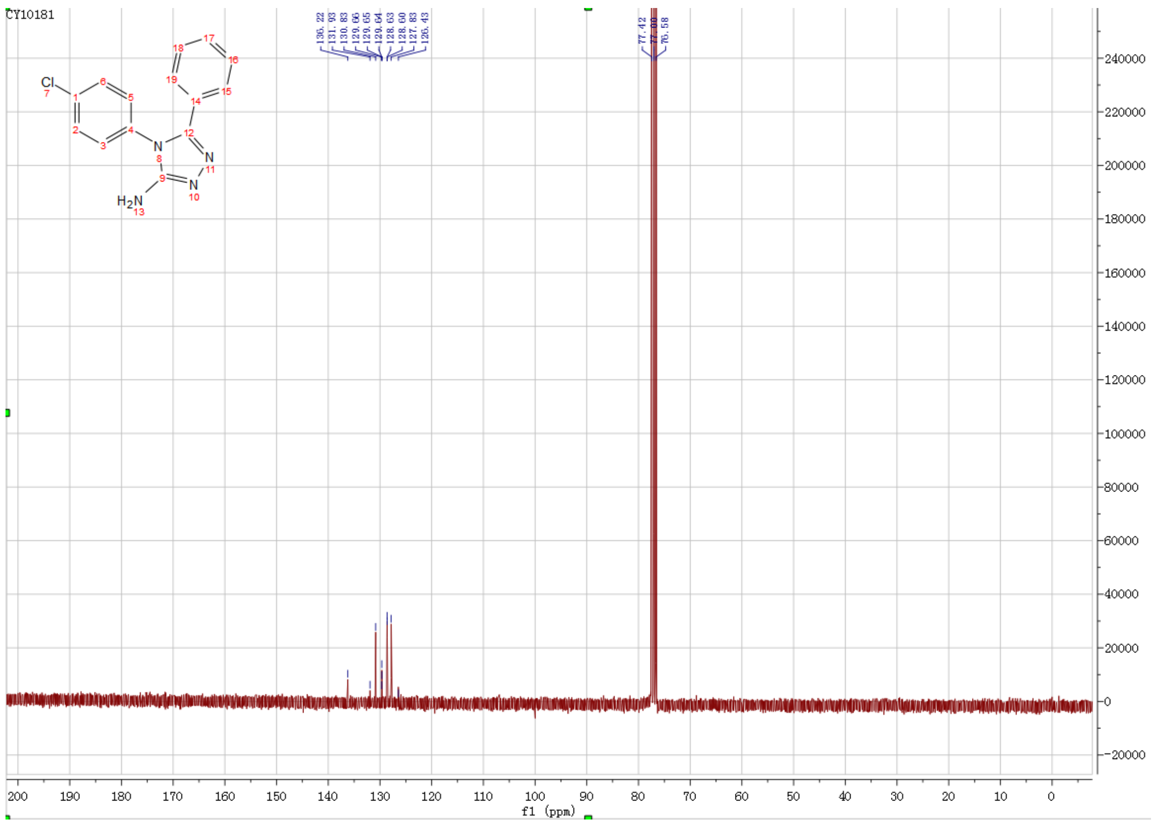

^{13}C-NMR of 4-(4-chlorophenyl)-5-phenyl-4*H*-1,2,4-triazol-3-amine **(4a)**

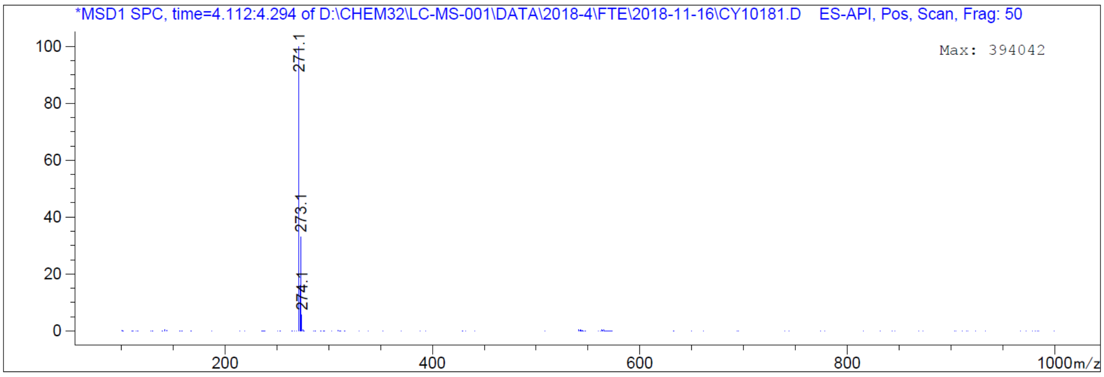

MS of 4-(4-chlorophenyl)-5-phenyl-4*H*-1,2,4-triazol-3-amine **(4a)**

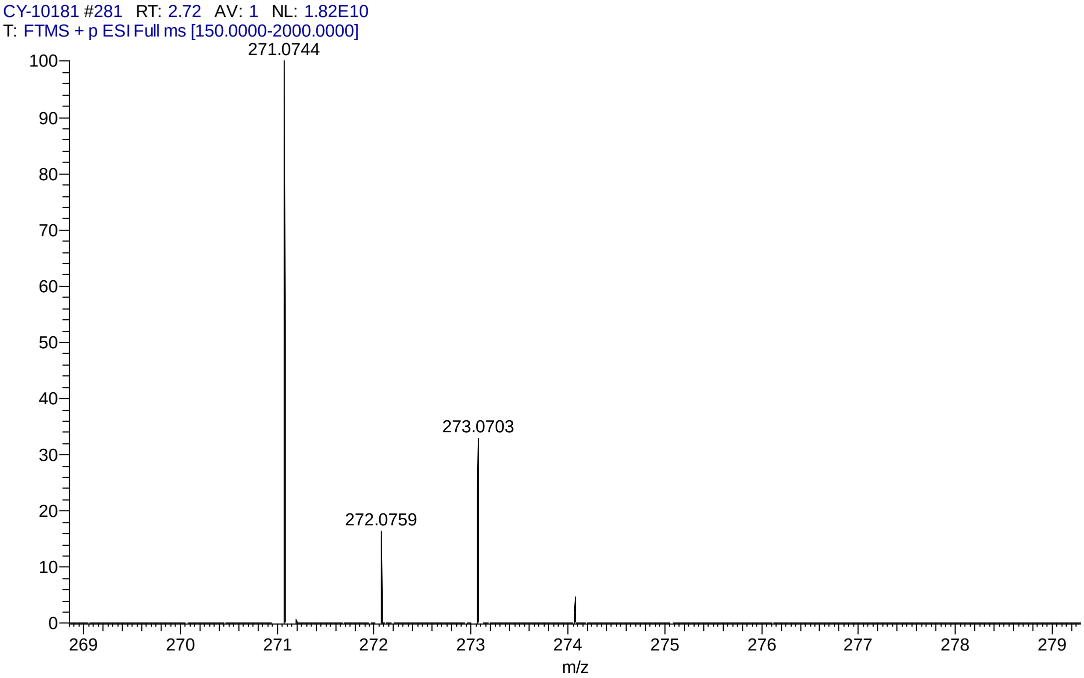

HRMS of 4-(4-chlorophenyl)-5-phenyl-4*H*-1,2,4-triazol-3-amine **(4a)**

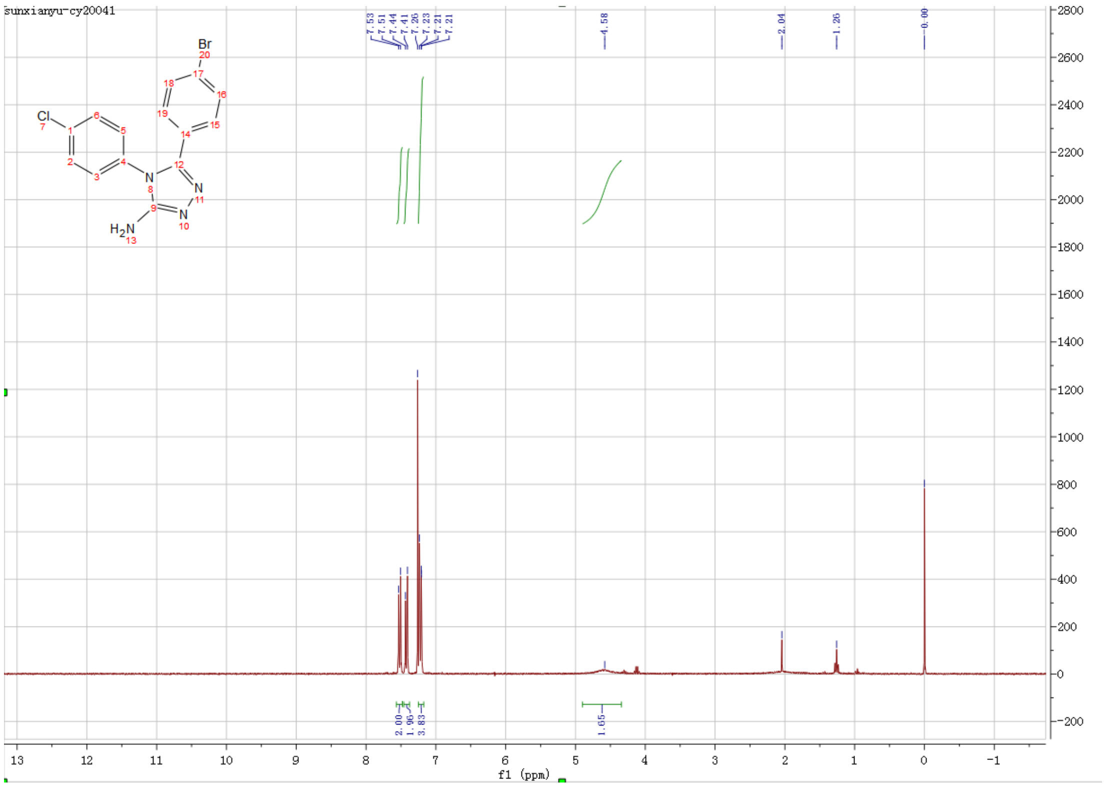

^{1}H-NMR of 5-(4-bromophenyl)-4-(4-chlorophenyl)-4*H*-1,2,4-triazol-3-amine **(4b)**

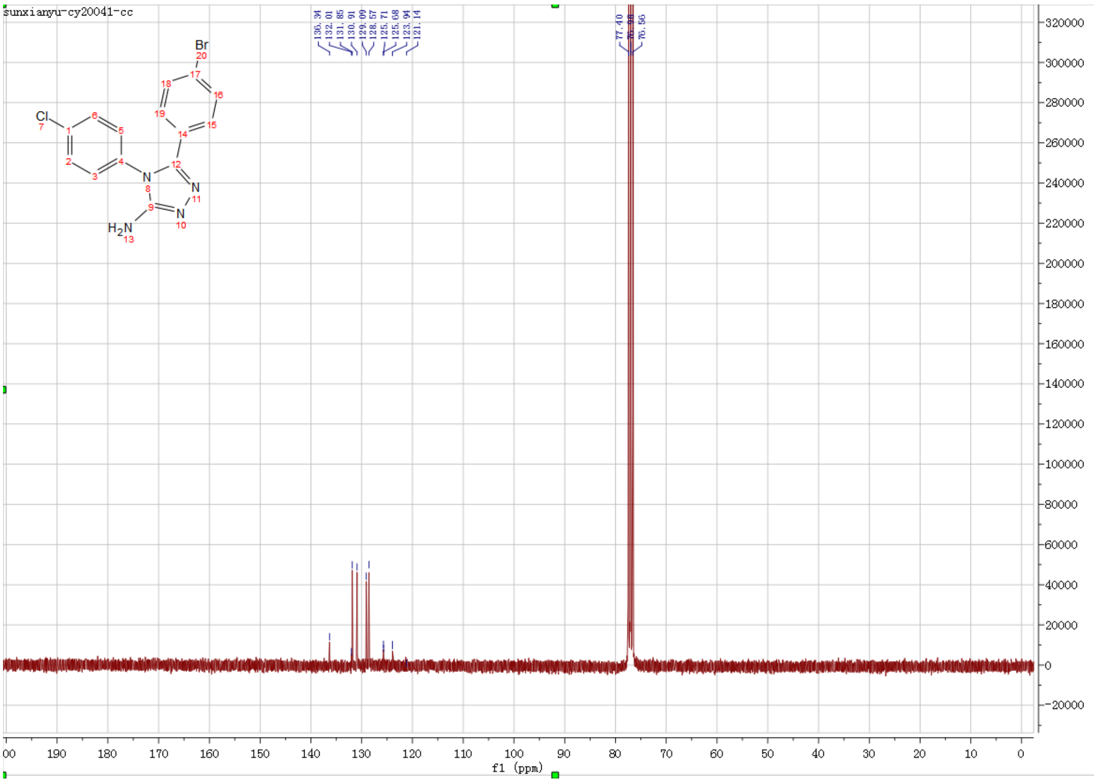

^{13}C-NMR of 5-(4-bromophenyl)-4-(4-chlorophenyl)-4H-1,2,4-triazol-3-amine **(4b)**

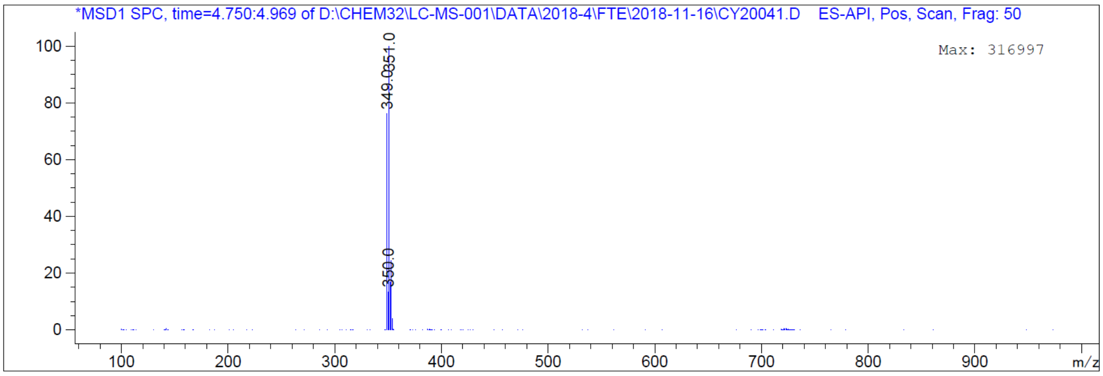

MS of 5-(4-bromophenyl)-4-(4-chlorophenyl)-4H-1,2,4-triazol-3-amine **(4b)**

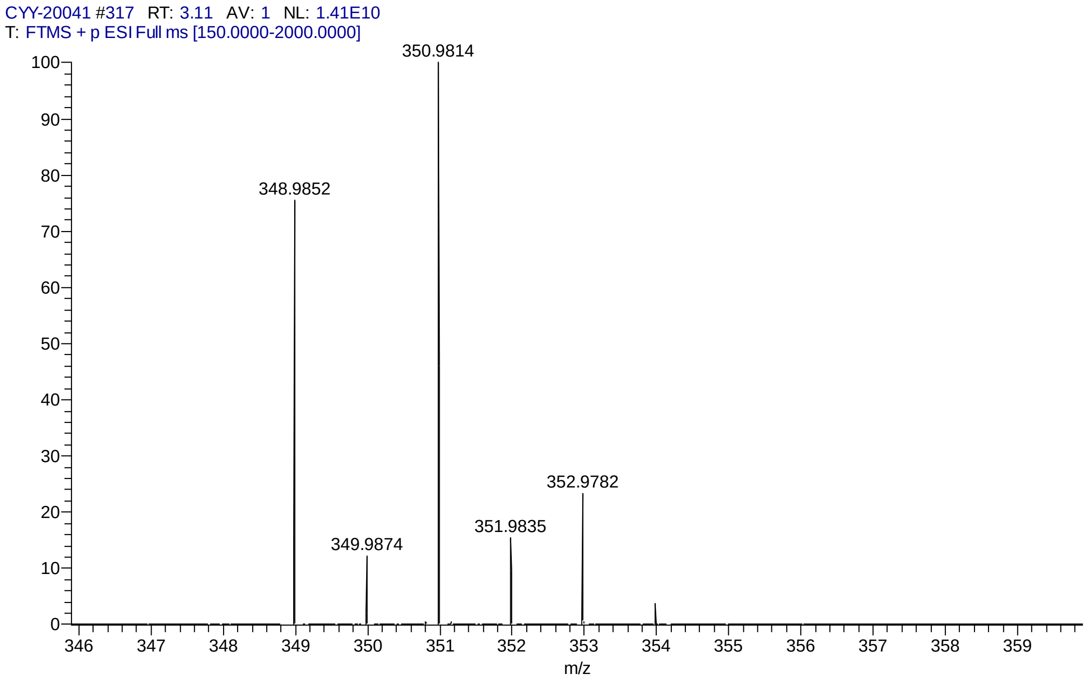

HRMS of 5-(4-bromophenyl)-4-(4-chlorophenyl)-4*H*-1,2,4-triazol-3-amine **(4b)**

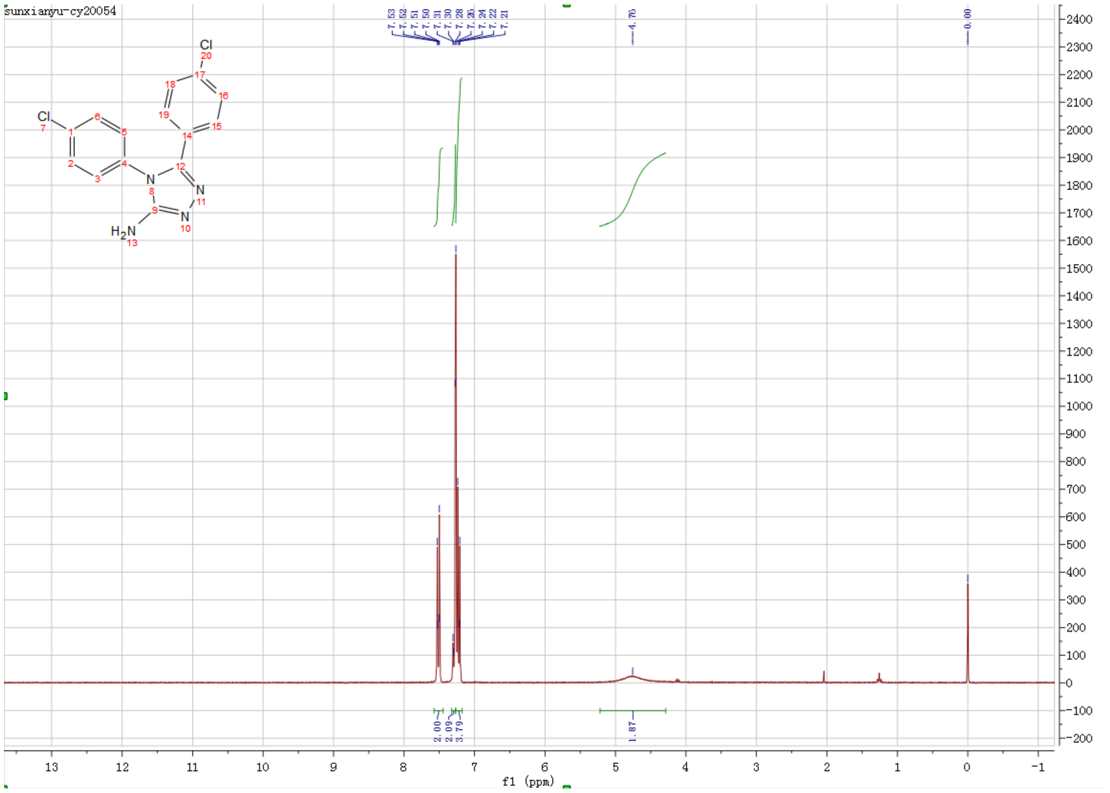

^{1}H-NMR of 4,5-bis(4-chlorophenyl)-4*H*-1,2,4-triazol-3-amine **(4c)**

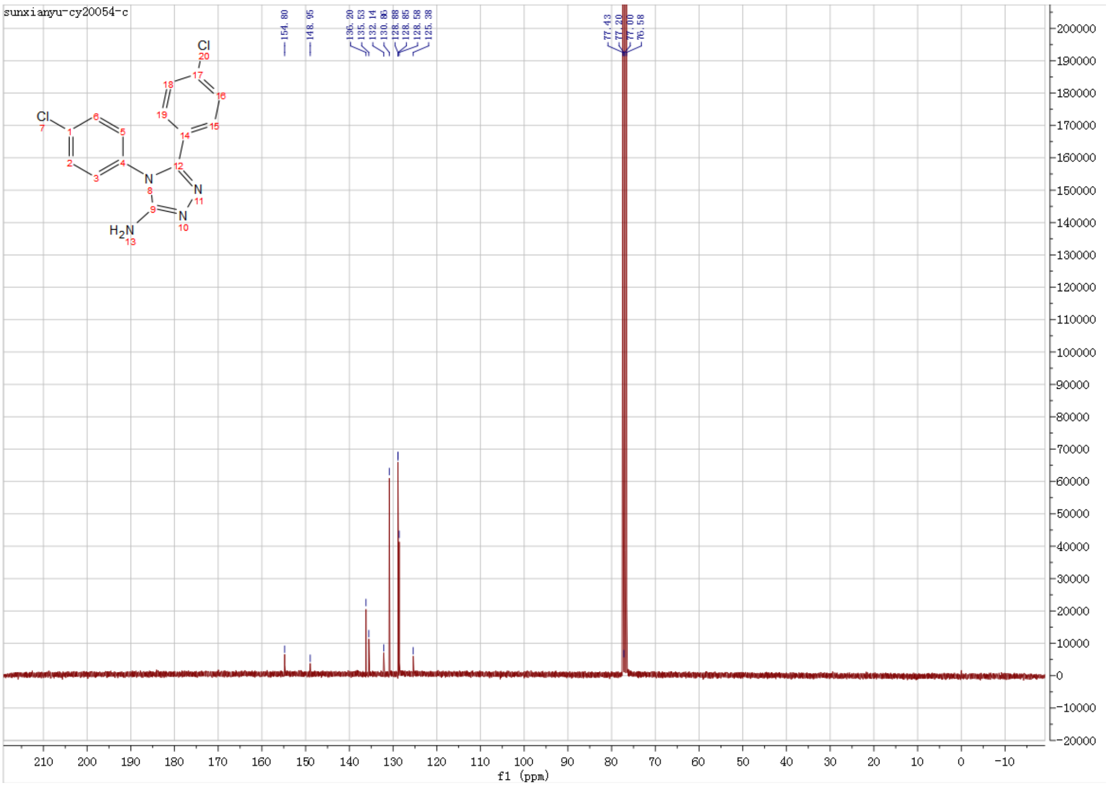

^{13}C-NMR of 4,5-bis(4-chlorophenyl)-4*H*-1,2,4-triazol-3-amine **(4c)**

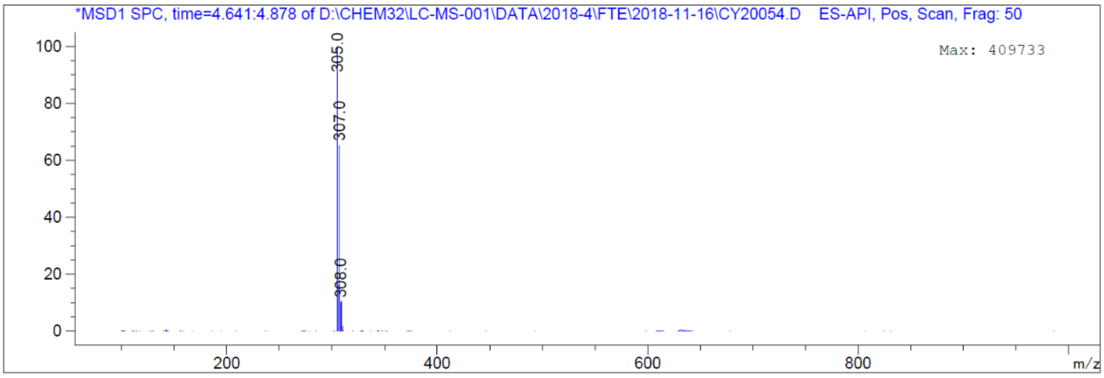

MS of 4,5-bis(4-chlorophenyl)-4*H*-1,2,4-triazol-3-amine **(4c)**

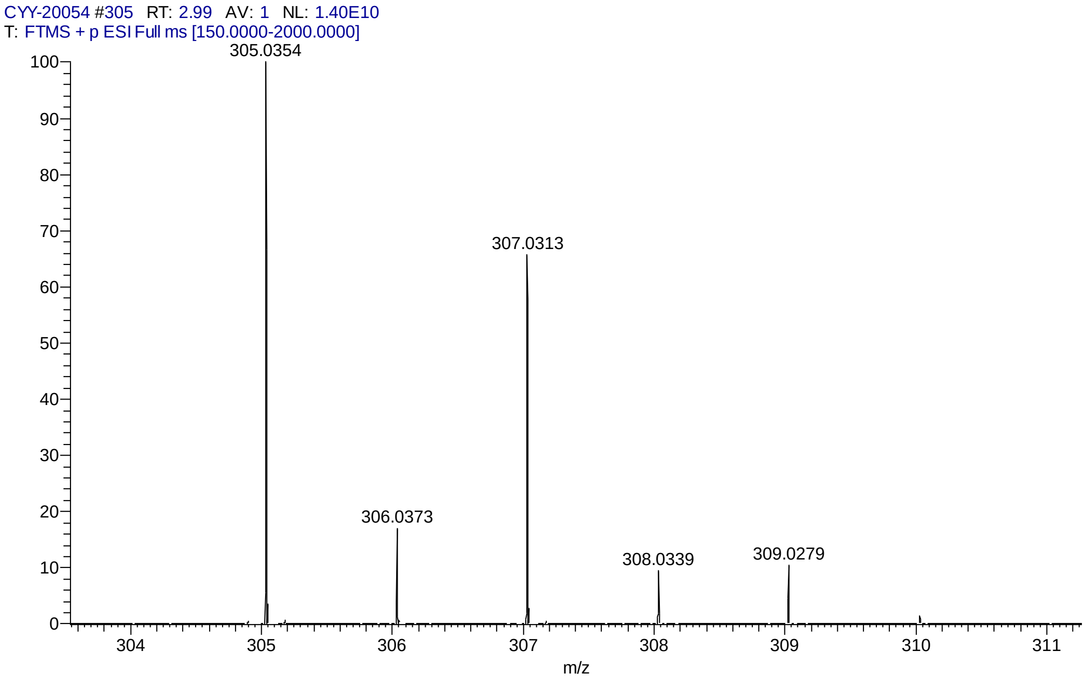

HRMS of 4,5-bis(4-chlorophenyl)-4*H*-1,2,4-triazol-3-amine **(4c)**

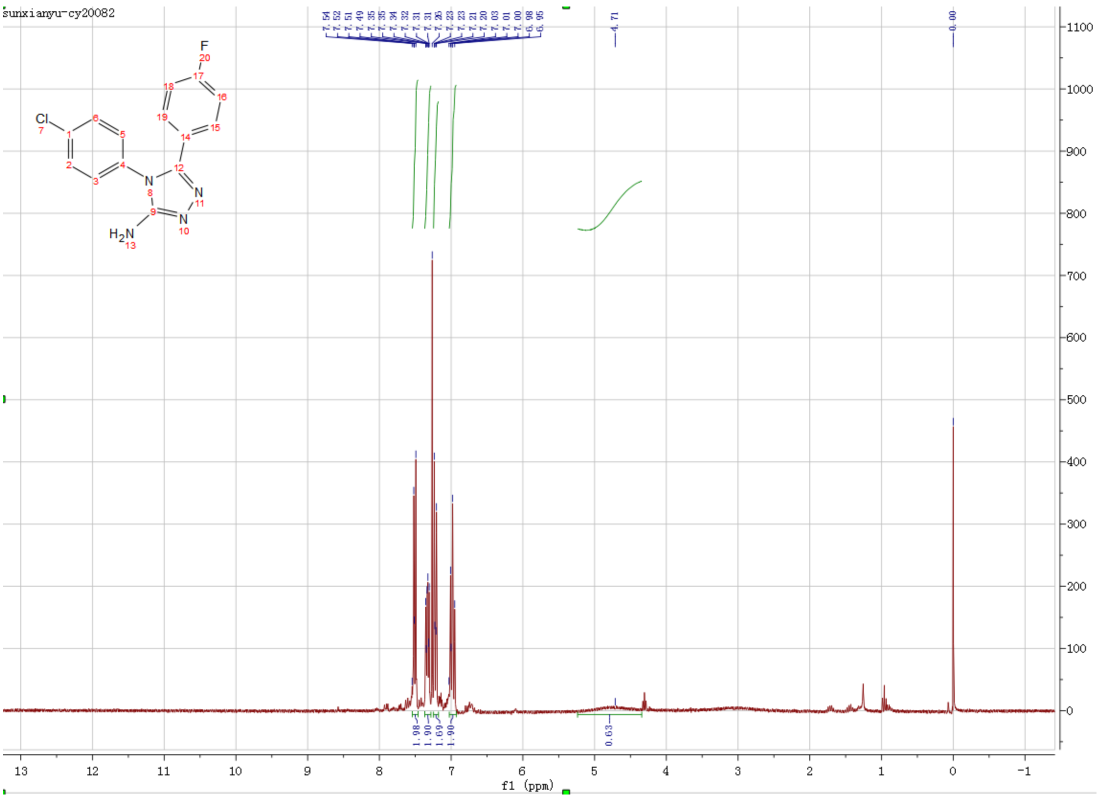

^{1}H-NMR of 4-(4-chlorophenyl)-5-(4-fluorophenyl)-4*H*-1,2,4-triazol-3-amine **(4d)**

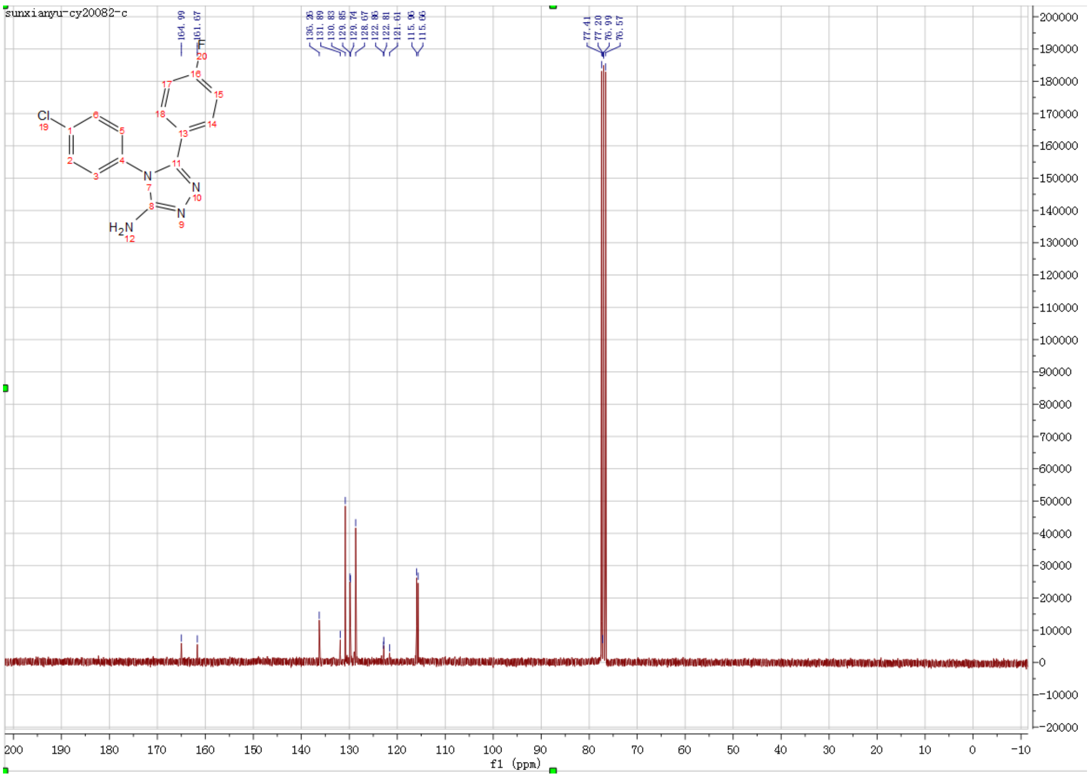

^{13}C-NMR of 4-(4-chlorophenyl)-5-(4-fluorophenyl)-4*H*-1,2,4-triazol-3-amine **(4d)**

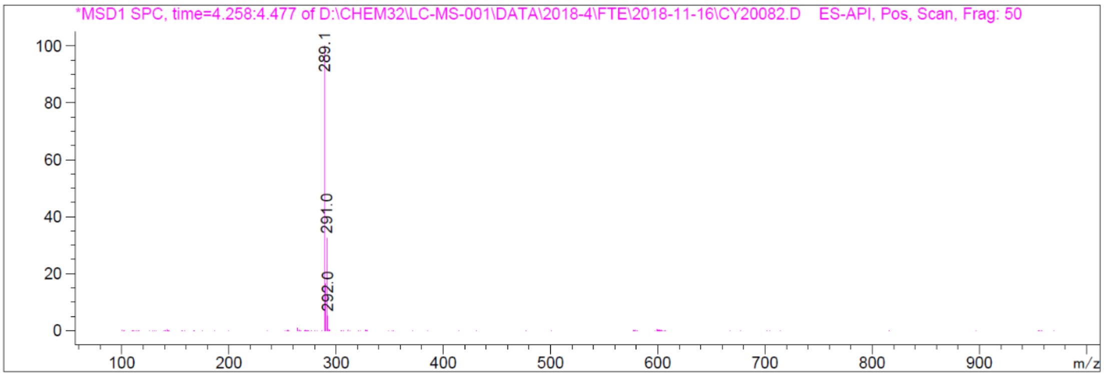

MS of 4-(4-chlorophenyl)-5-(4-fluorophenyl)-4*H*-1,2,4-triazol-3-amine **(4d)**

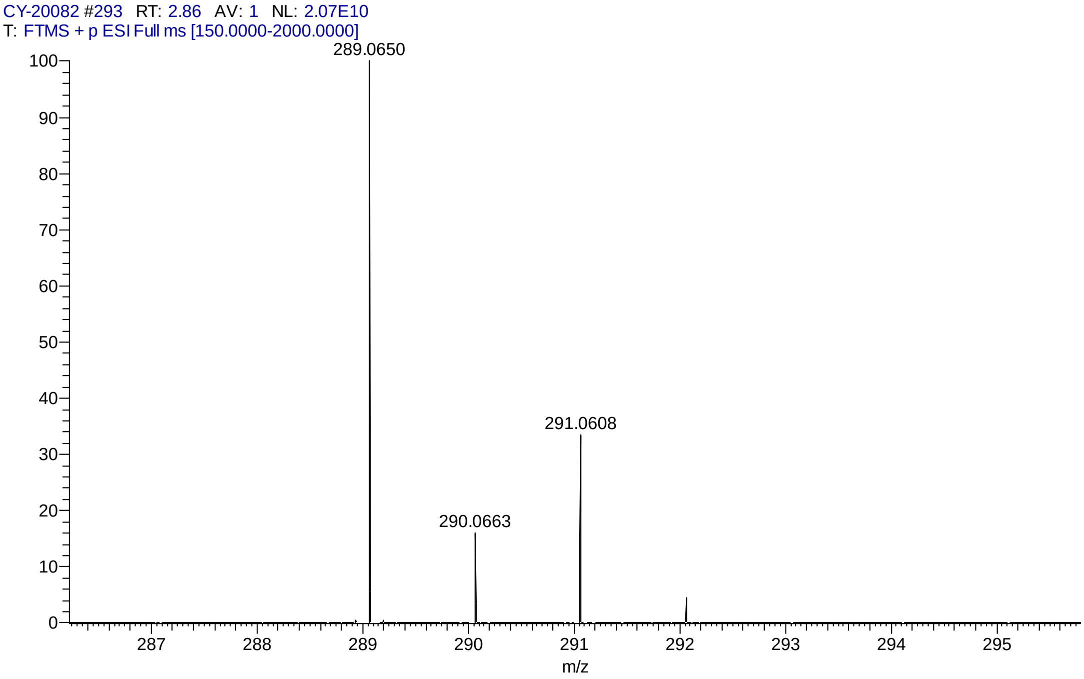

HRMS of 4-(4-chlorophenyl)-5-(4-fluorophenyl)-4*H*-1,2,4-triazol-3-amine **(4d)**

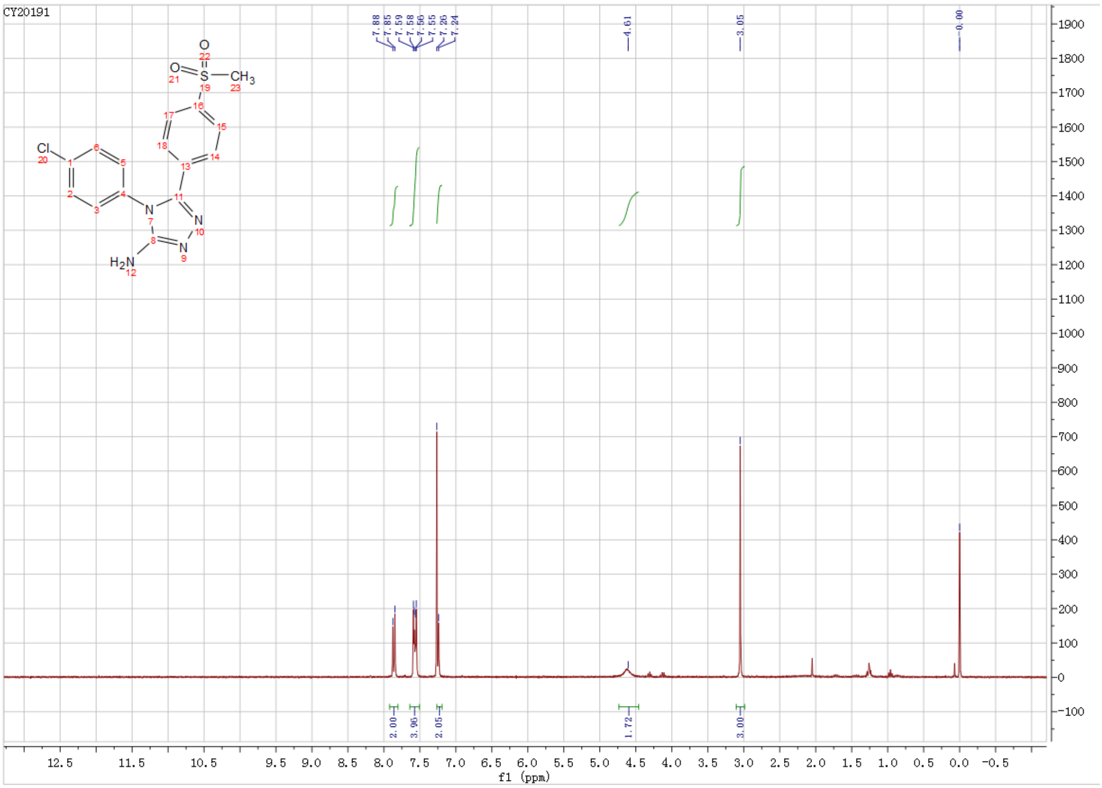

^{1}H-NMR of 4-(4-chlorophenyl)-5-[4-(methylsulfonyl)phenyl]-4*H*-1,2,4-triazol-3-amine **(4e)**

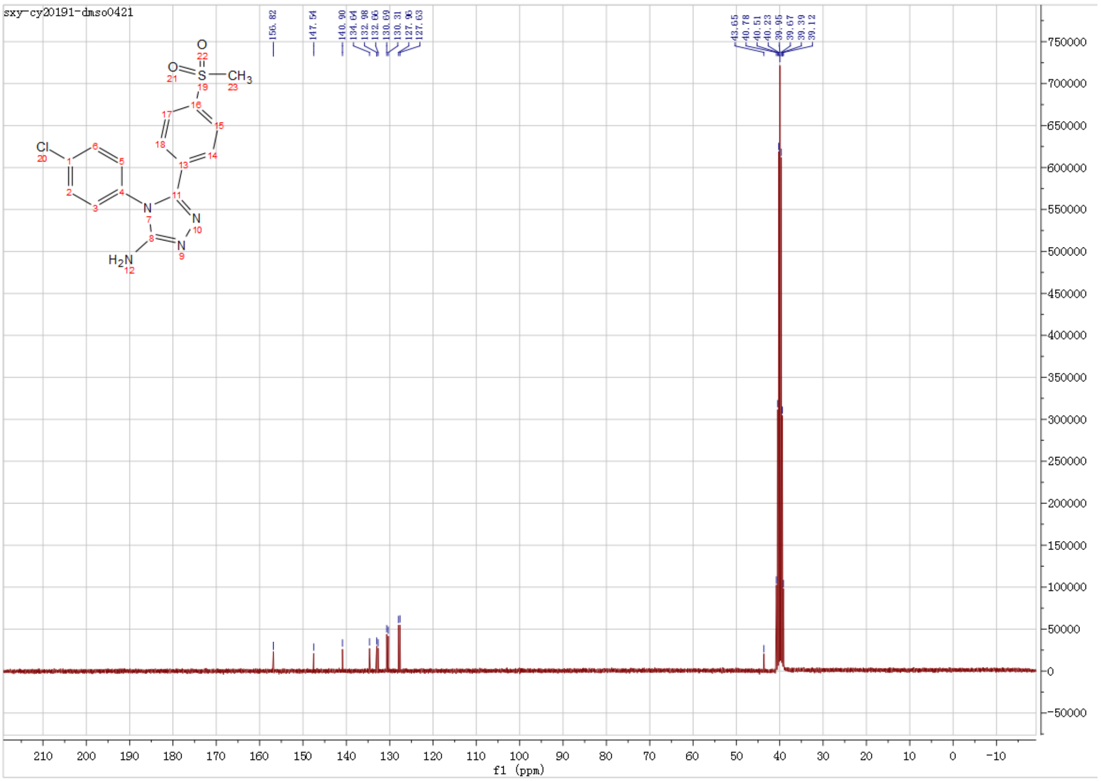

^{13}C-NMR of 4-(4-chlorophenyl)-5-[4-(methylsulfonyl)phenyl]-4*H*-1,2,4-triazol-3-amine **(4e)**

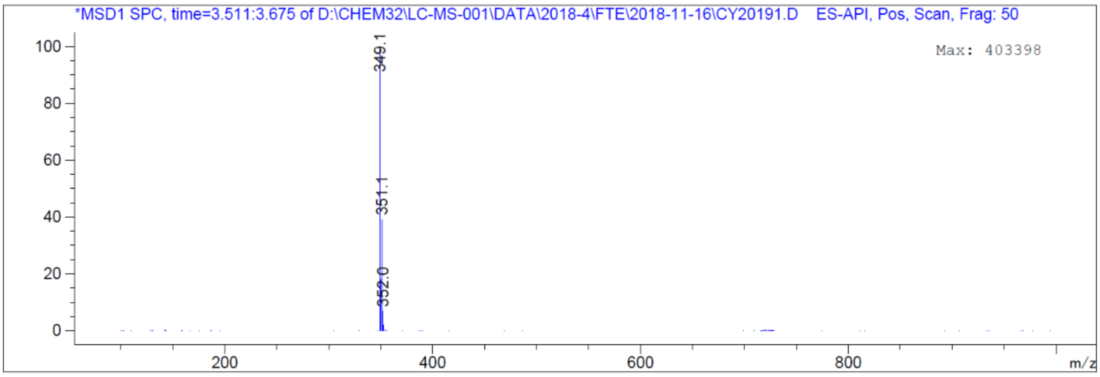

MS of 4-(4-chlorophenyl)-5-[4-(methylsulfonyl)phenyl]-4*H*-1,2,4-triazol-3-amine **(4e)**

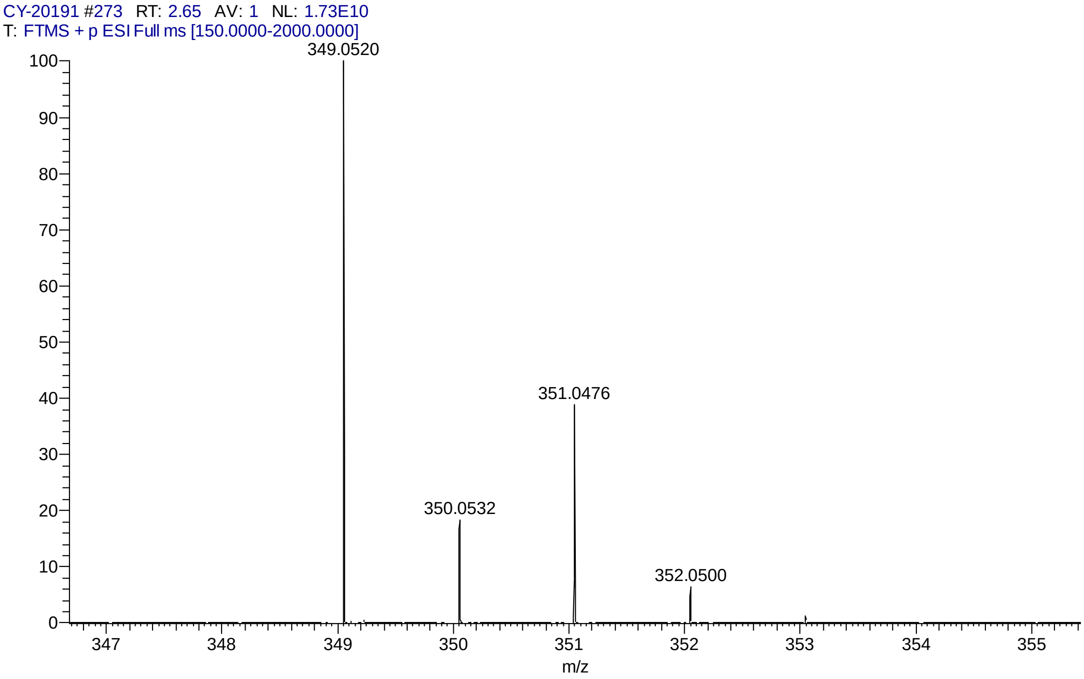

HRMS of 4-(4-chlorophenyl)-5-[4-(methylsulfonyl)phenyl]-4*H*-1,2,4-triazol-3-amine **(4e)**

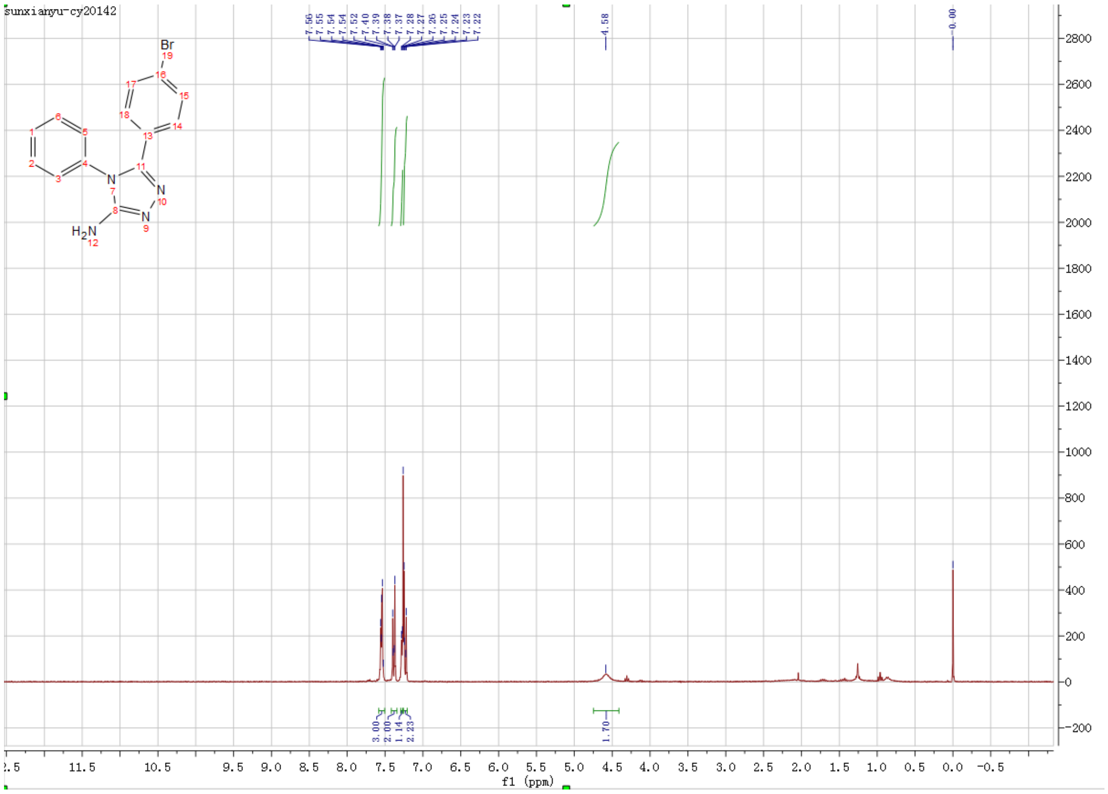

^{1}H-NMR of 5-(4-bromophenyl)-4-phenyl-4*H*-1,2,4-triazol-3-amine **(4f)**

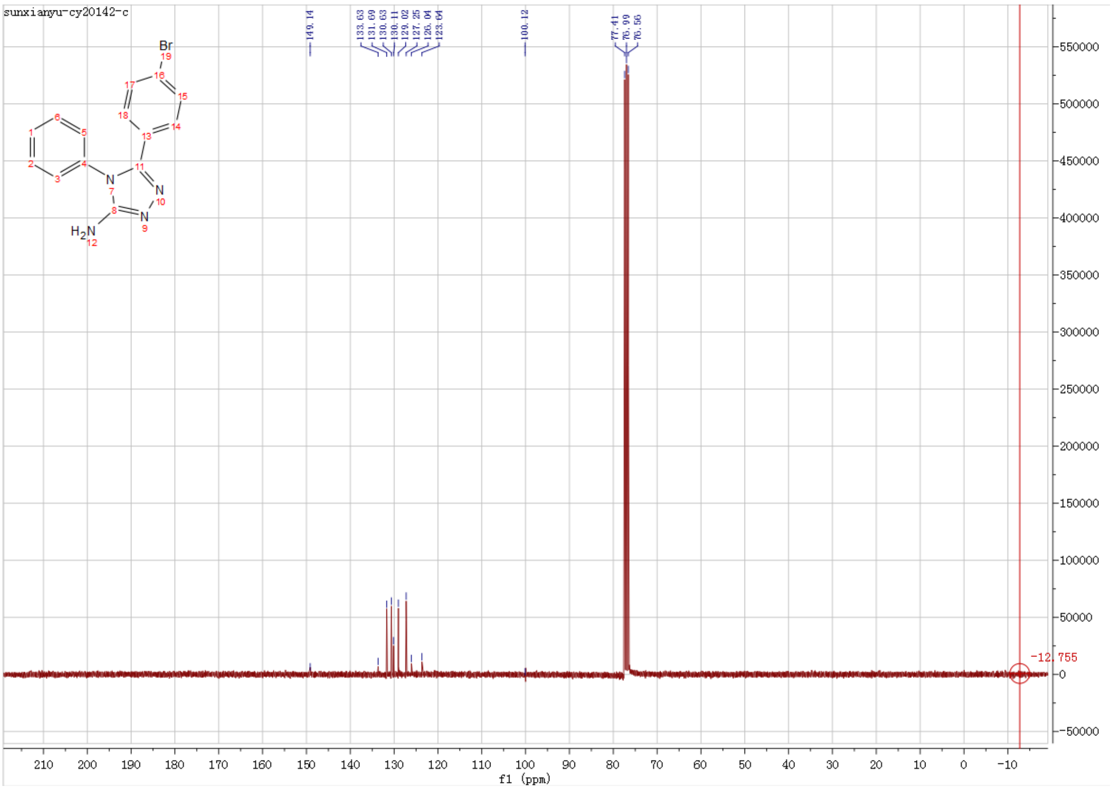

^{13}C-NMR of 5-(4-bromophenyl)-4-phenyl-4*H*-1,2,4-triazol-3-amine **(4f)**

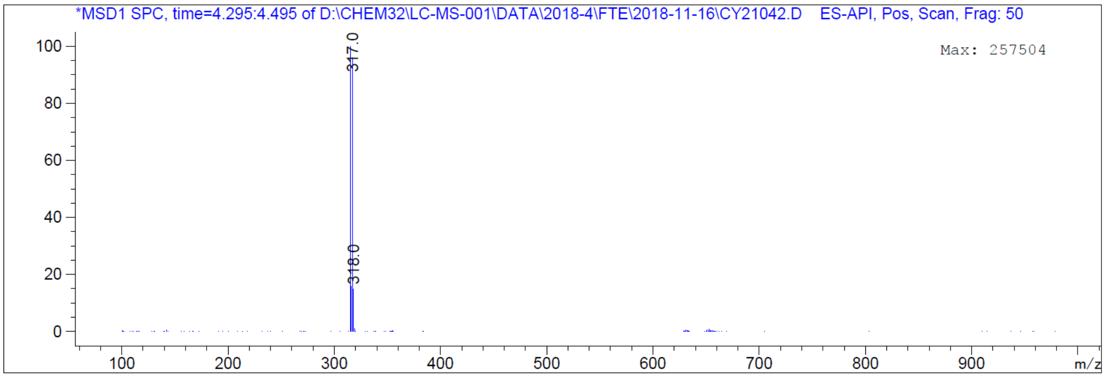

MS of 5-(4-bromophenyl)-4-phenyl-4*H*-1,2,4-triazol-3-amine **(4f)**

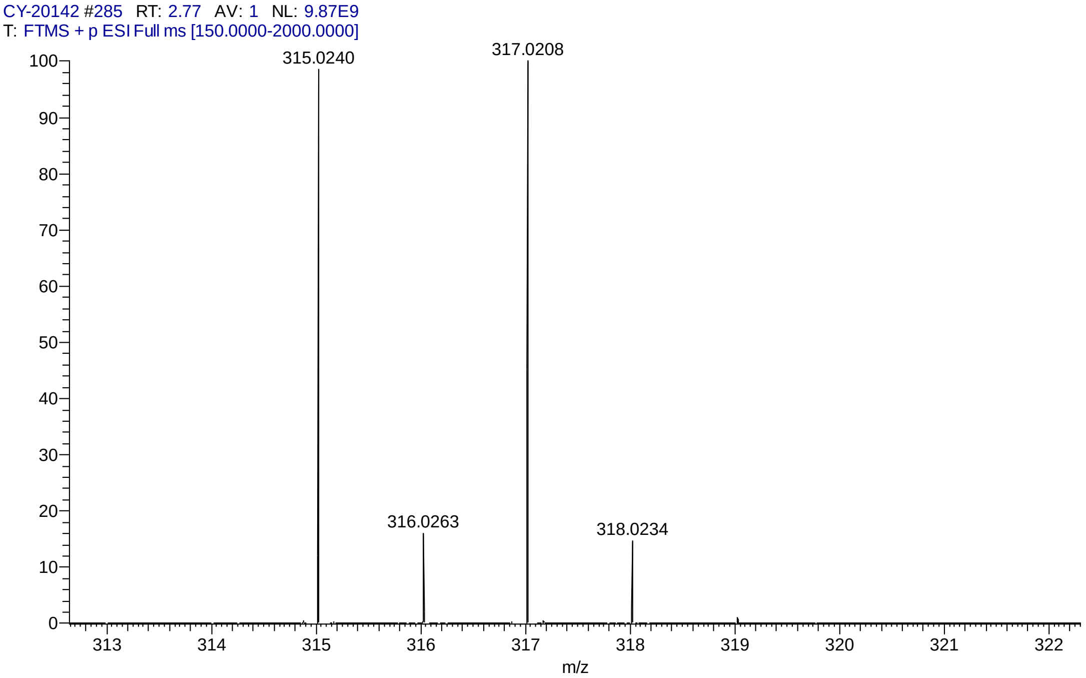

HRMS of 5-(4-bromophenyl)-4-phenyl-4*H*-1,2,4-triazol-3-amine **(4f)**

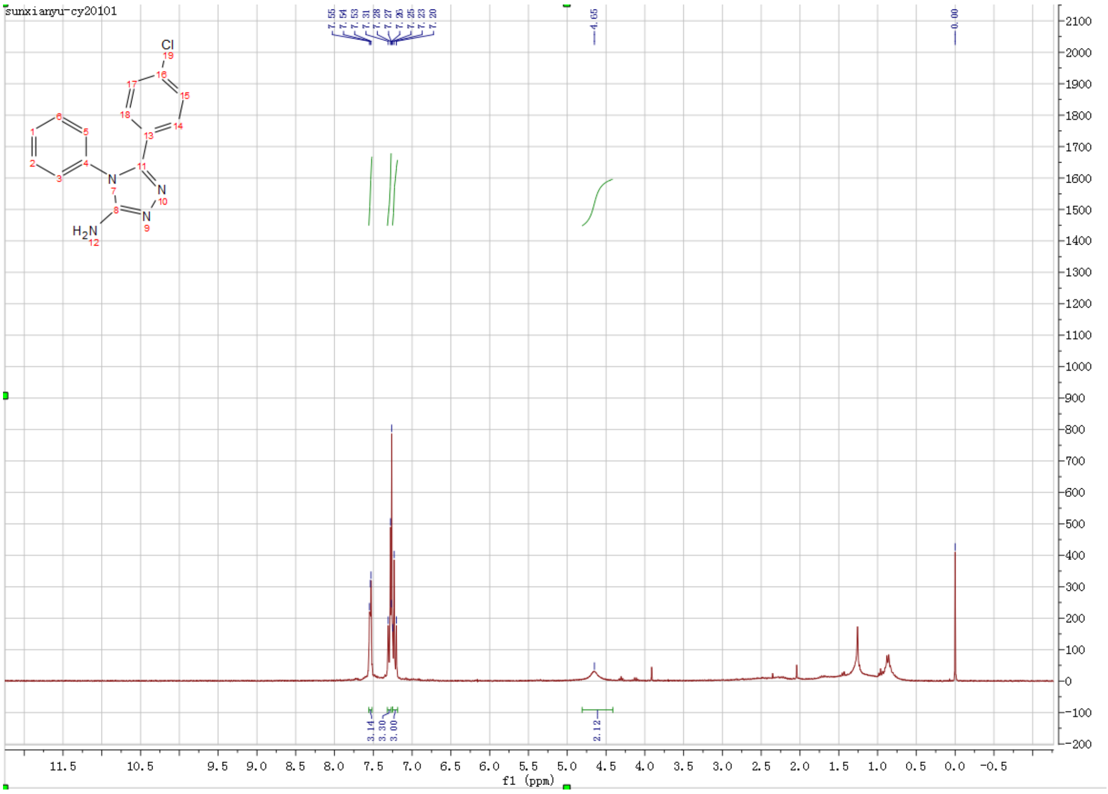

^{1}H-NMR of 5-(4-chlorophenyl)-4-phenyl-4*H*-1,2,4-triazol-3-amine **(4g)**

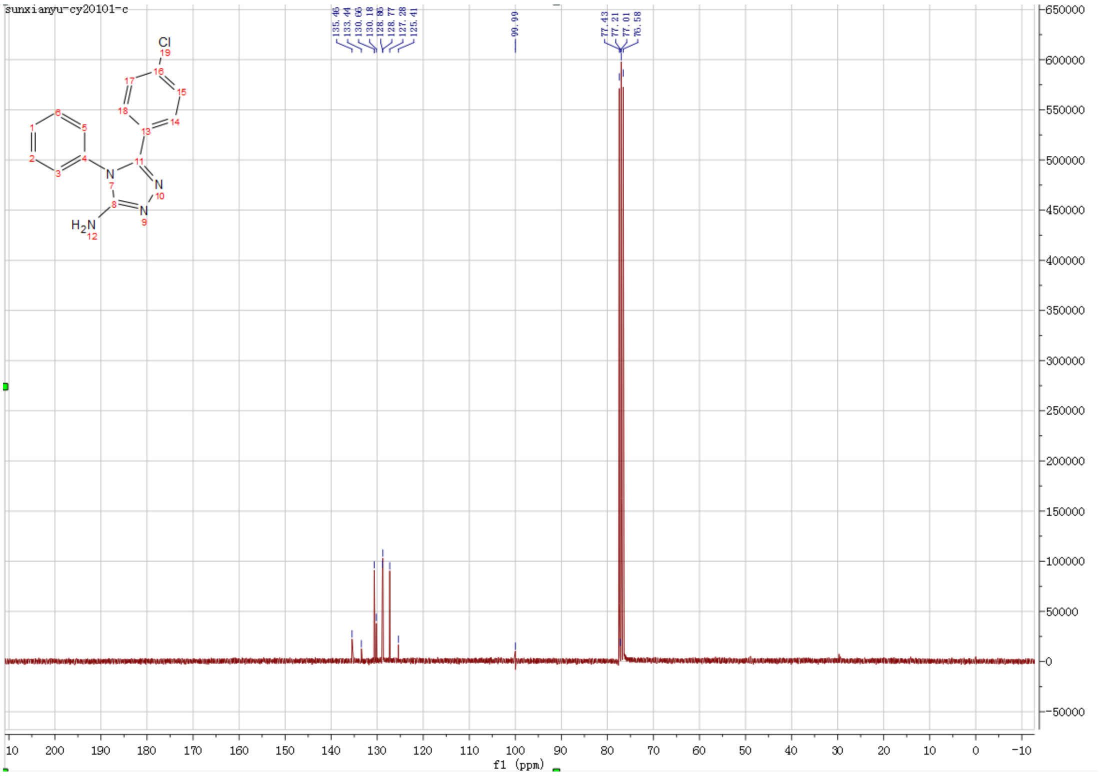

^{13}C-NMR of 5-(4-chlorophenyl)-4-phenyl-4*H*-1,2,4-triazol-3-amine **(4g)**

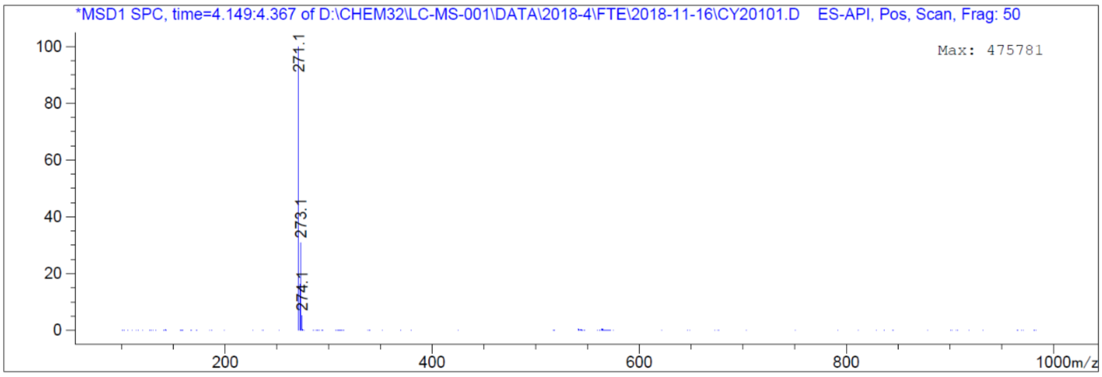

MS of 5-(4-chlorophenyl)-4-phenyl-4*H*-1,2,4-triazol-3-amine **(4g)**

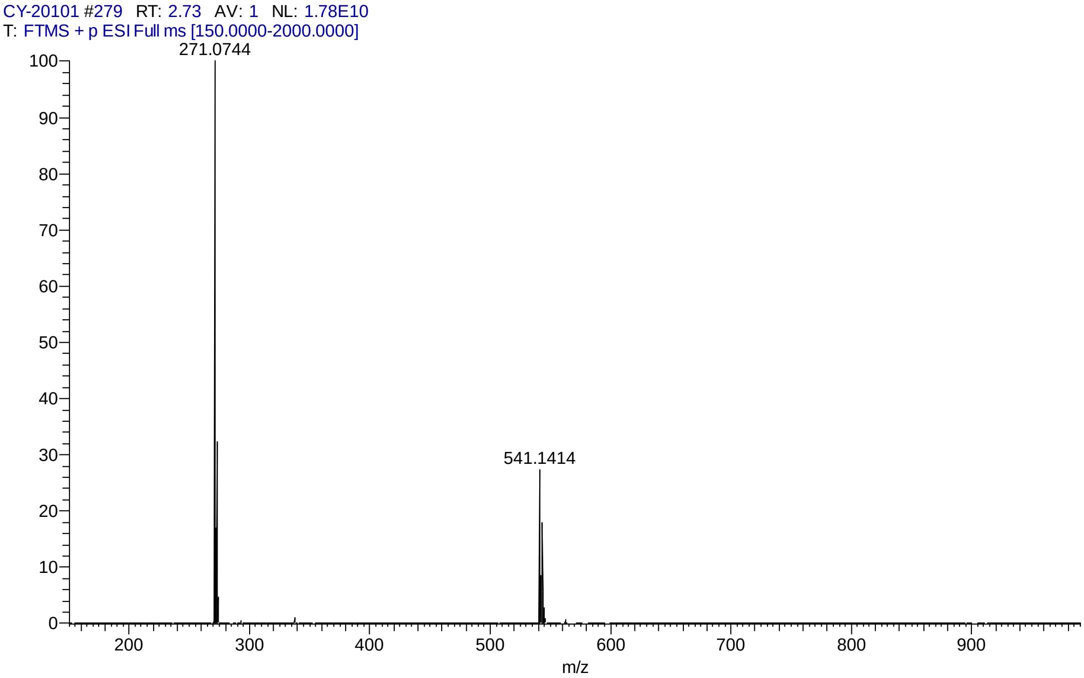

HRMS of 5-(4-chlorophenyl)-4-phenyl-4*H*-1,2,4-triazol-3-amine **(4g)**

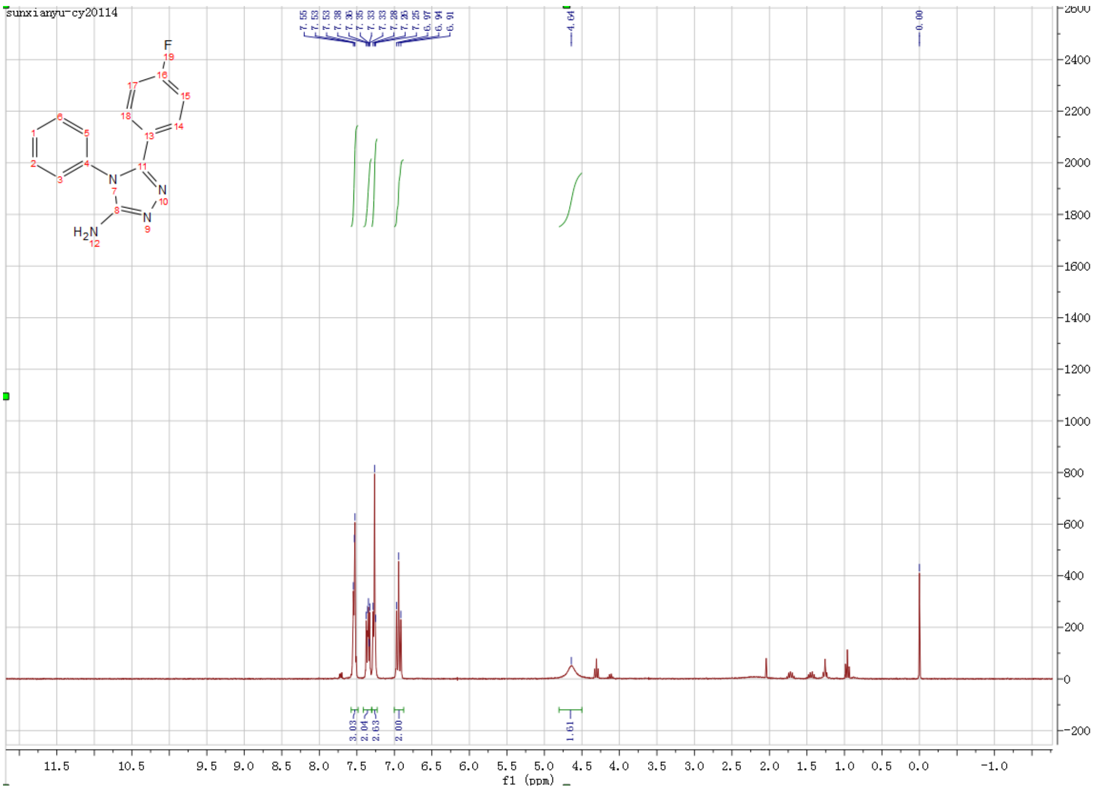

^{1}H-NMR of 5-(4-fluorophenyl)-4-phenyl-4*H*-1,2,4-triazol-3-amine **(4h)**

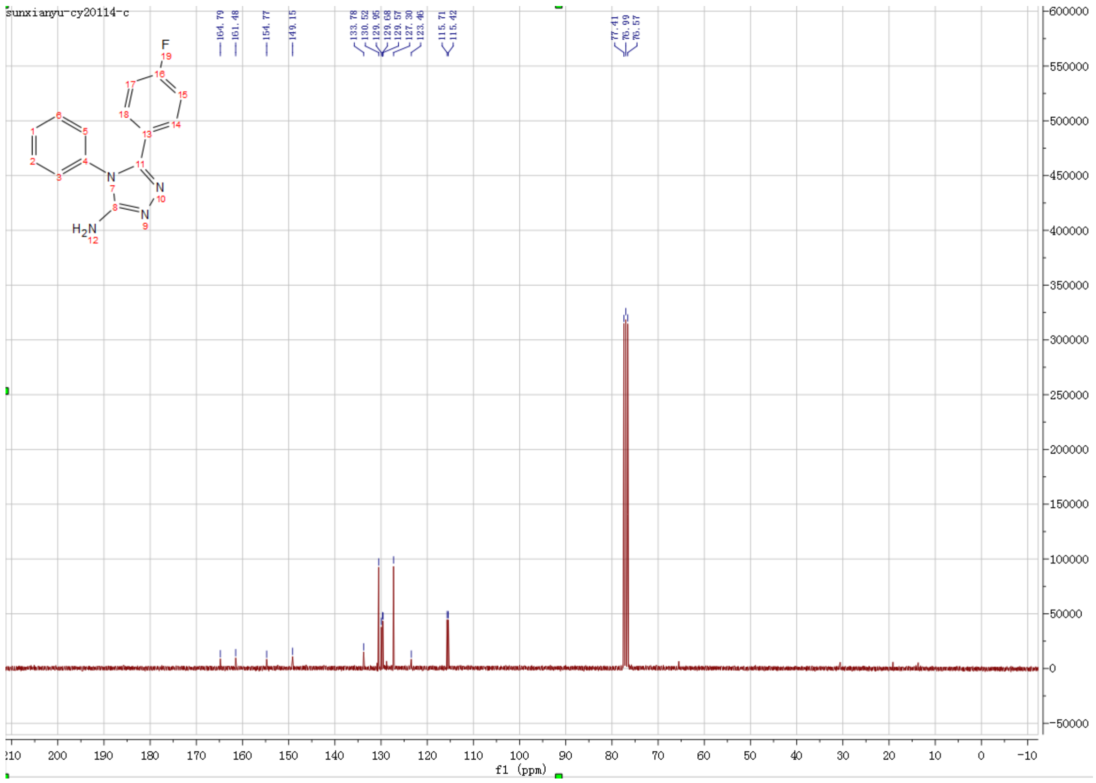

^{13}C-NMR of 5-(4-fluorophenyl)-4-phenyl-4*H*-1,2,4-triazol-3-amine **(4h)**

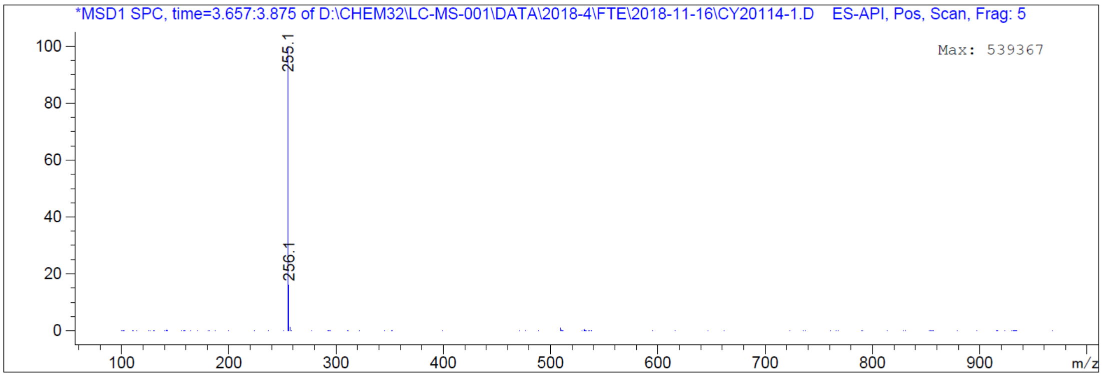

MS of 5-(4-fluorophenyl)-4-phenyl-4*H*-1,2,4-triazol-3-amine **(4h)**

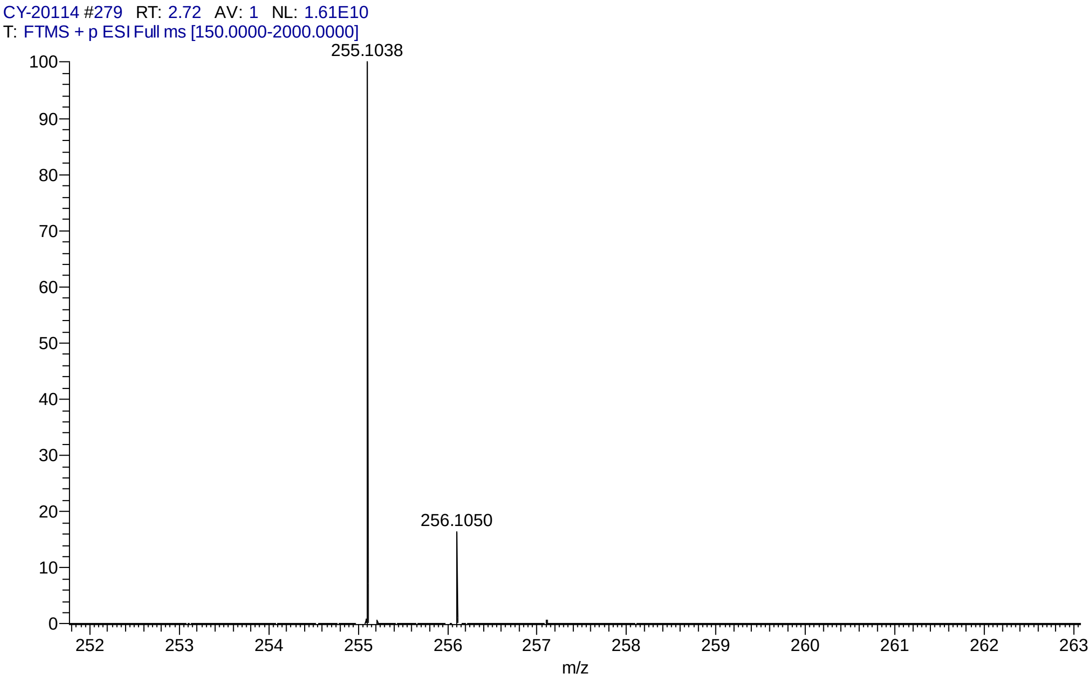

HRMS of 5-(4-fluorophenyl)-4-phenyl-4*H*-1,2,4-triazol-3-amine **(4h)**

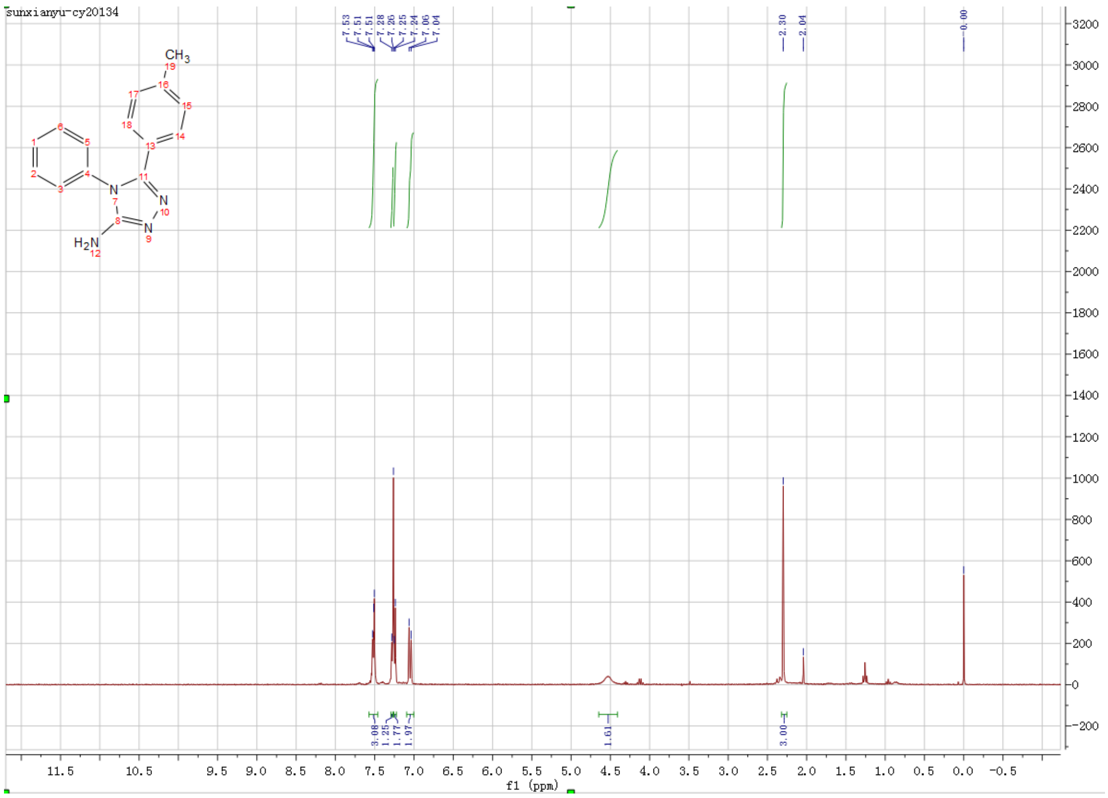

^{1}H-NMR of 4-phenyl-5-p-tolyl-4*H*-1,2,4-triazol-3-amine **(4i)**

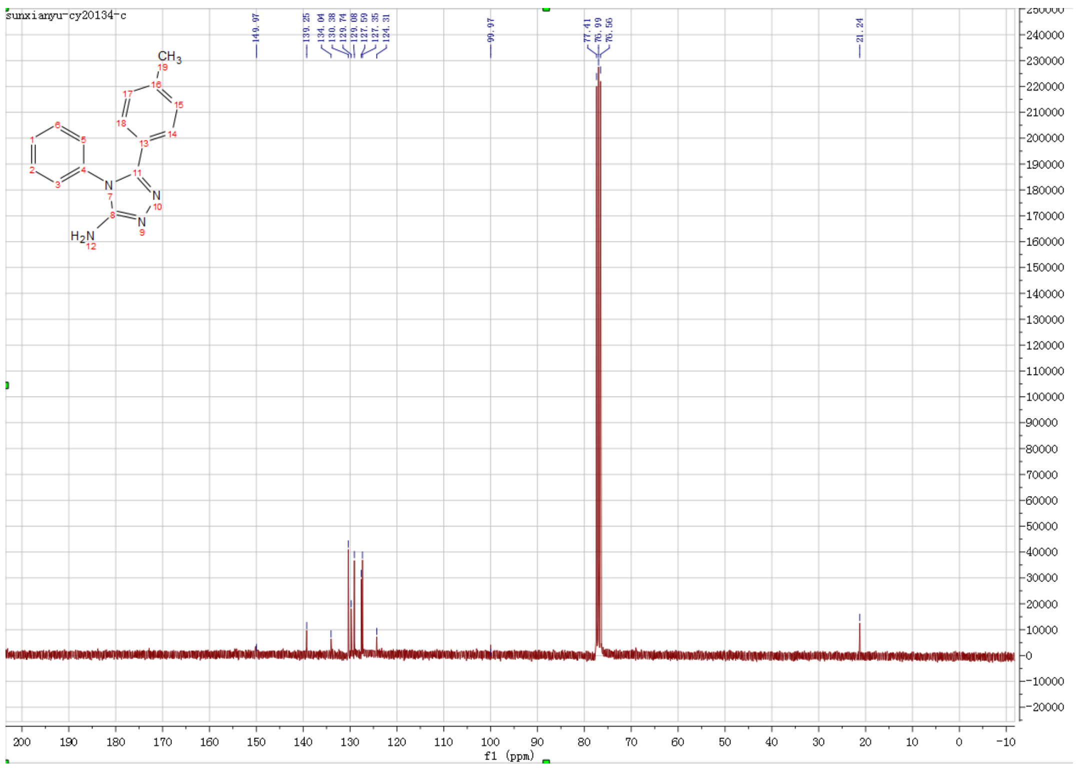

^{13}C-NMR of 4-phenyl-5-p-tolyl-4*H*-1,2,4-triazol-3-amine **(4i)**

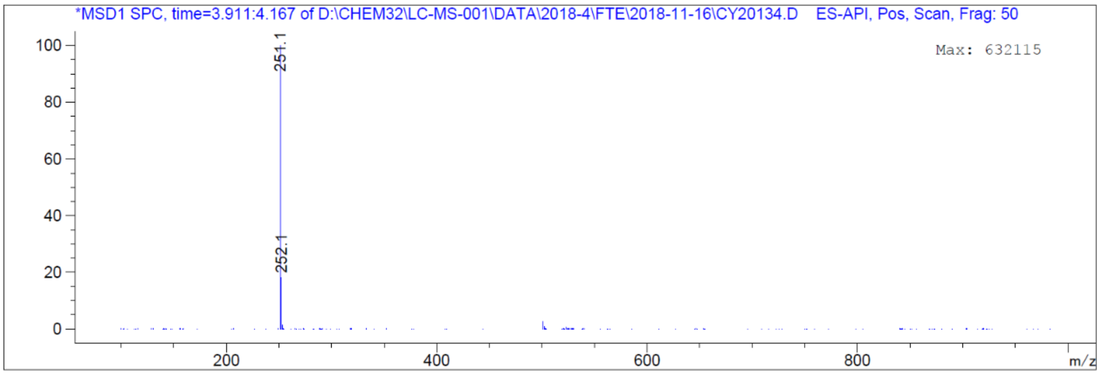

MS of 4-phenyl-5-p-tolyl-4*H*-1,2,4-triazol-3-amine **(4i)**

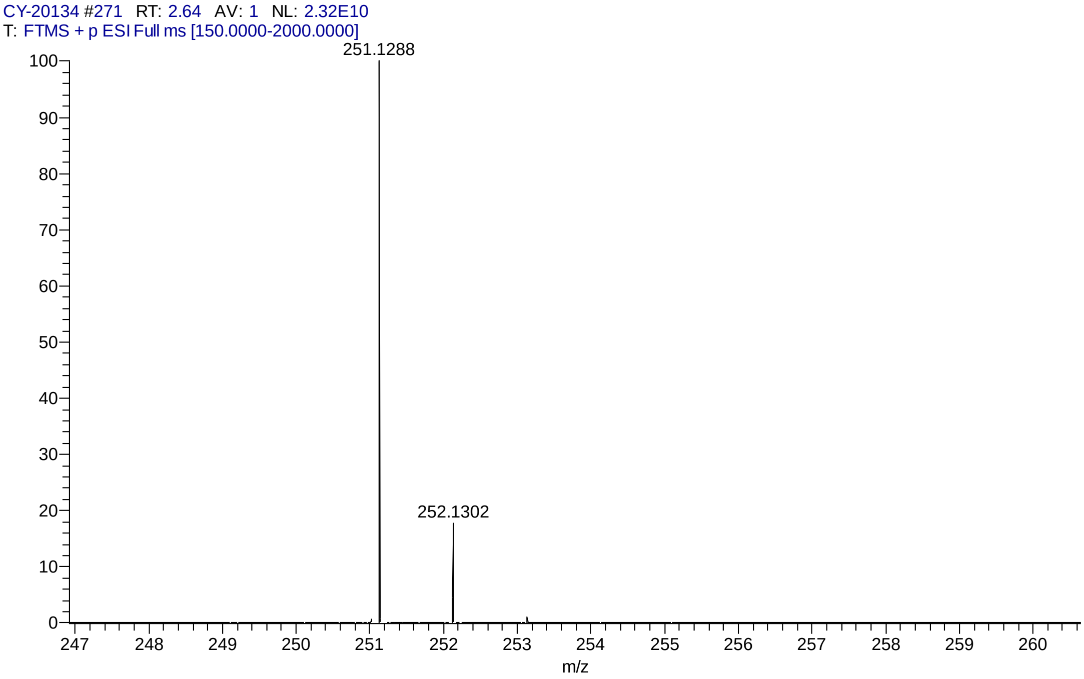

HRMS of 4-phenyl-5-p-tolyl-4*H*-1,2,4-triazol-3-amine **(4i)**

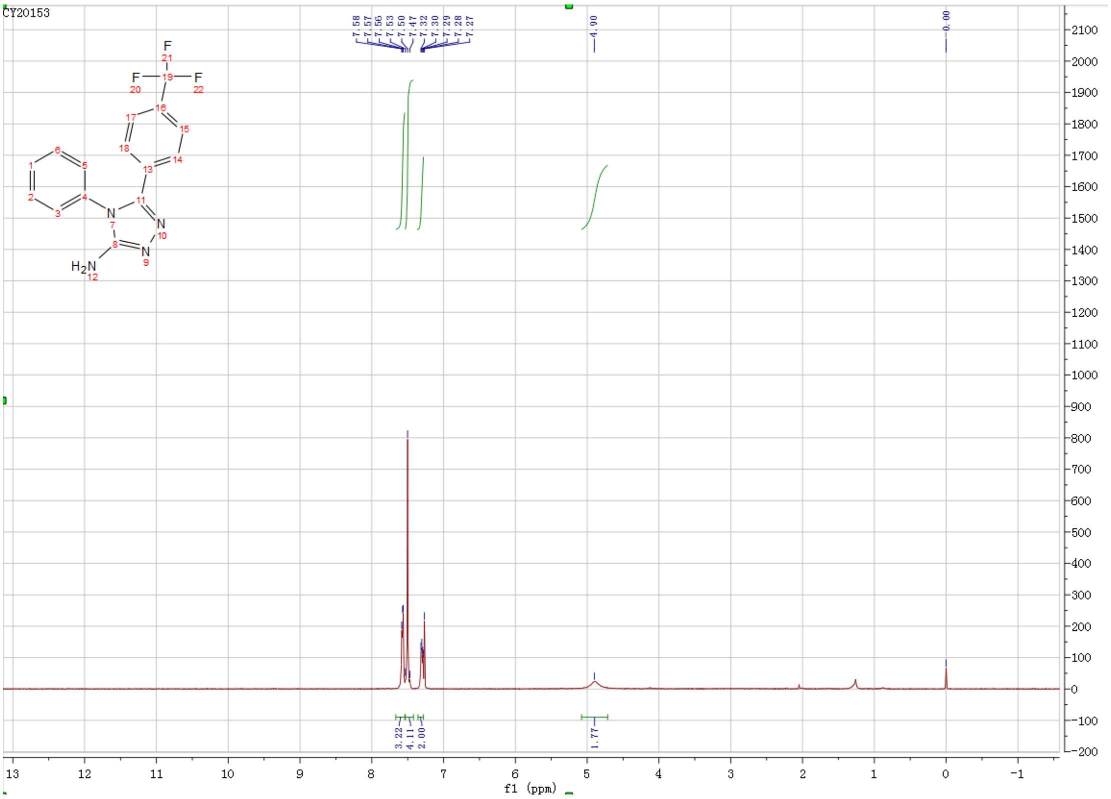

^{1}H-NMR of 5-(4-(trifluoromethyl)phenyl)-4-phenyl-4*H*-1,2,4-triazol-3-amine **(4i)**

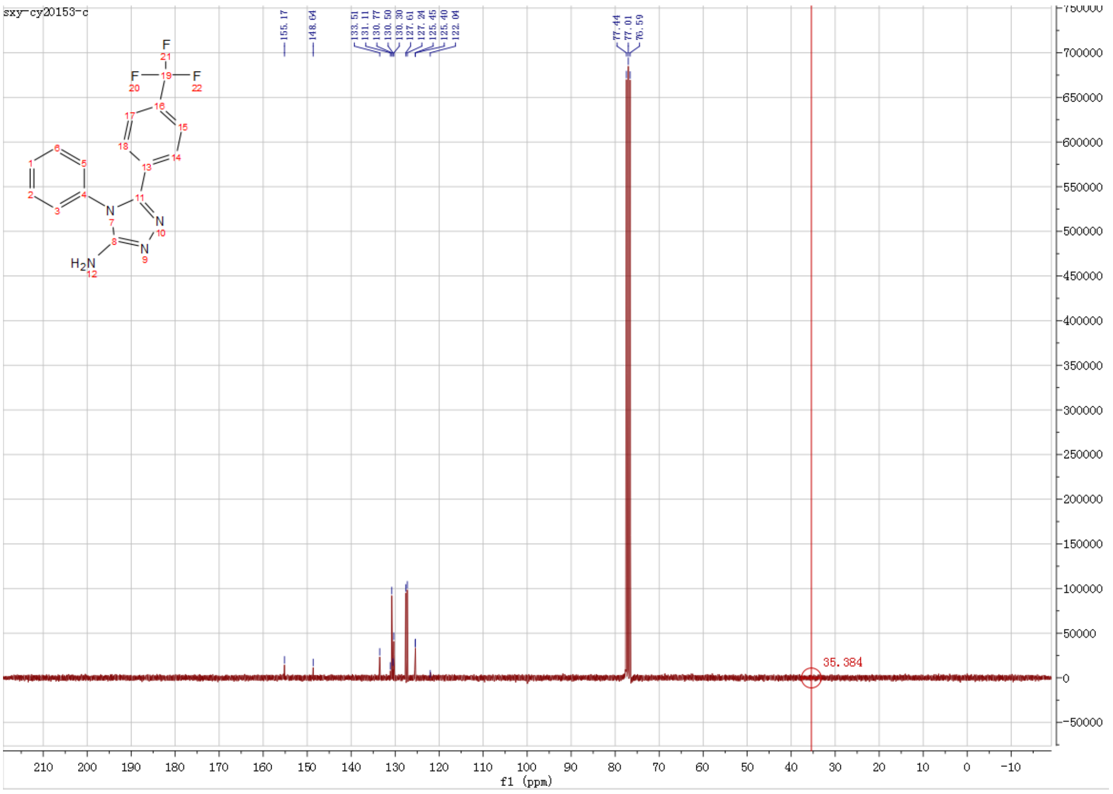

^{13}C-NMR of 5-(4-(trifluoromethyl)phenyl)-4-phenyl-4*H*-1,2,4-triazol-3-amine **(4i)**

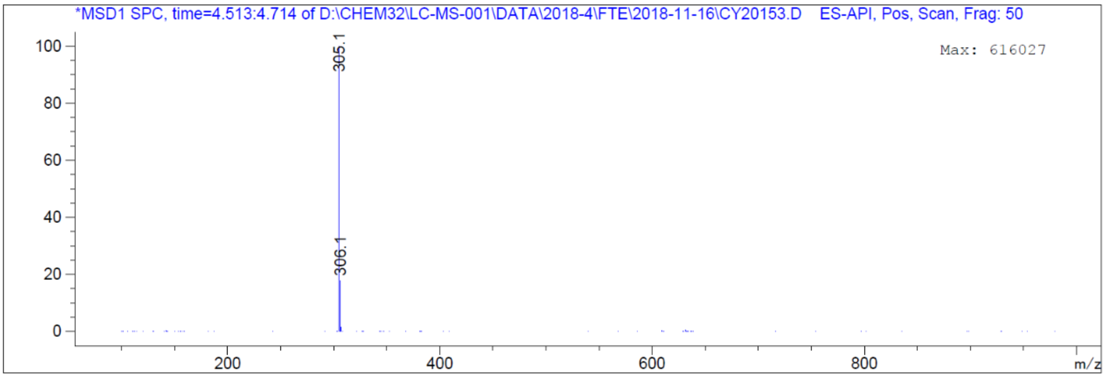

MS of 5-(4-(trifluoromethyl)phenyl)-4-phenyl-4*H*-1,2,4-triazol-3-amine **(4i)**

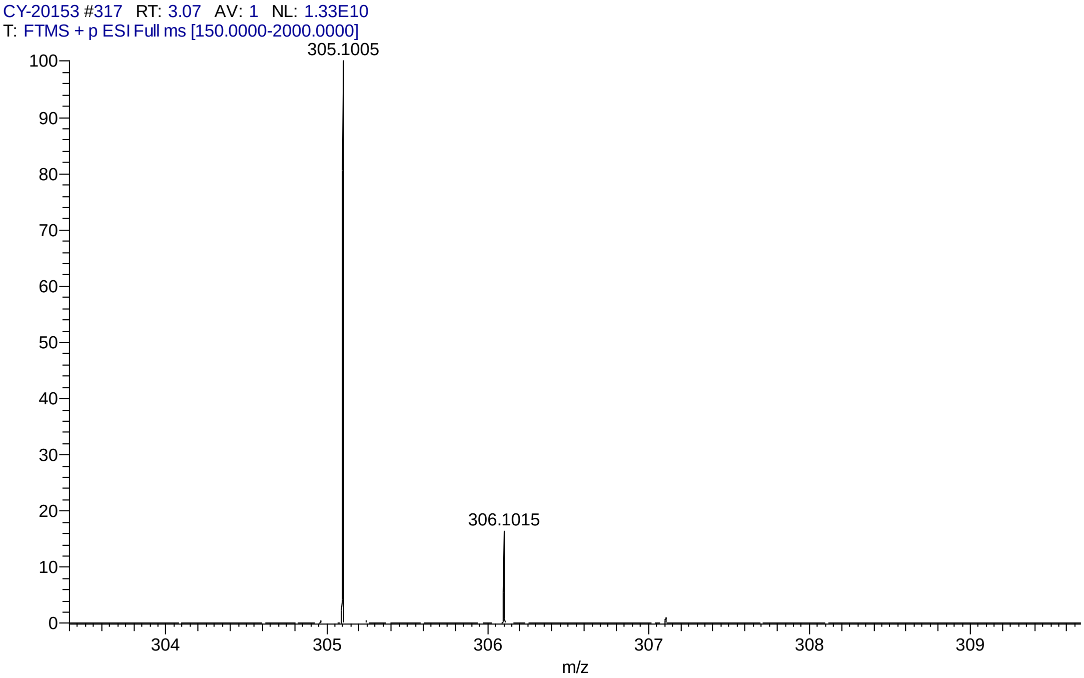

HRMS of 5-(4-(trifluoromethyl)phenyl)-4-phenyl-4*H*-1,2,4-triazol-3-amine **(4i)**
